# Energy planning in Sub-Saharan African countries needs to explicitly consider productive uses of electricity

**DOI:** 10.1038/s41598-023-40021-y

**Published:** 2023-08-10

**Authors:** Anteneh G. Dagnachew, Su-Min Choi, Giacomo Falchetta

**Affiliations:** 1 PBL Netherlands Environmental Assessment Agency, The Hague, The Netherlands; 2 Copernicus Institute of Sustainable Development, Utrecht University, Utrecht, The Netherlands; 3 International Institute for Applied Systems Analysis, Laxenburg, Austria; 4 Centro Euro-Mediterraneo sui Cambiamenti Climatici (CMCC), Venice, Italy

**Keywords:** Energy access, Energy supply and demand

## Abstract

Studies show the role of various electrification technologies in providing electricity access to households in Sub-Saharan Africa, with a focus on electricity demand for end-use services such as lighting, cooking, heating, cooling and other appliance use. The demand for productive use of electricity, which is important to enhance income generation opportunities and labour productivity, is usually not considered. Using the IMAGE-TIMER integrated assessment model framework, we present a methodology to project the impact of productive activities on the electricity system of the region. We show that growing productive activities increase household electricity demand by half, which has important consequences for determining the cost-optimal electrification technologies. We argue that planning of electricity systems should accommodate this increase in electricity demand for productive uses. In addition, while productive uses of electricity have a positive impact on the financial viability of electrification systems, they also increase the electricity sector investment requirements considerably.

## Introduction

In recent years, some countries in Sub-Saharan Africa (SSA) have significantly improved access to electricity, a key development priority and a pillar of Sustainable Development Goal 7. The region’s population without access has decreased from 610 million in 2013 to 567.5 million in 2020^1^, despite the rapid population growth in the region. However, the number of people that lack access to electricity has increased to 590 million in 2021, as the progress of the past years was slowed down by the COVID pandemic^2^. Several recent studies^3–6^ have explored energy access challenges in SSA, and show that, at business-as-usual trends, over half a billion people would be left without access to electricity in 2030. Both off-grid and centralized grid systems need to be scaled up significantly to achieve universal access to electricity, requiring estimated annual investment needs in the range of 13–73 billion USD on top of baseline investments^4,7^.

These studies mostly consider household electricity demand for end-use services, i.e. lighting, space heating, space cooling, water heating, cooking, and other household appliance use. However, others^8,9^ have clearly pointed to the necessity of addressing the demand for productive use of electricity (PUE), which is important to enhance income generation opportunities and labour productivity. PUE is the additional electricity use of households on top of their consumption for end-use services in order to provide additional income through household enterprises. These activities include agricultural energy uses (such as water pumping for smallholder irrigation), agro-processing (i.e. milling, husking, hulling), small-scale manufacturing (e.g. carpentry, tailoring, welding, looming) and service sector (like gastronomy, beauty salon, etc.). Household enterprises are so small that they are often carried out inside the home of the entrepreneur entrepreneur and often result from a common decision making process of the household, rather than by individual members^10^. Only few of these enterprises get formal registration^11^ and nearly 90% of micro, small and medium enterprises employing 2–9 persons in SSA are informal.

Despite the high share of households engaging in agricultural activities in SSA, its contribution to GDP is low. Current crop processing procedures, such as grinding millet, rice, maize, and cassava for gruels and porridges, are mostly done with a conventional, time-consuming mortar and pestle^12^. Electrifying these processes would greatly improve productivity, product quality and increase value added, which would not only increase household income but also relieve individuals from time and labour-intensive activities. At the same time, amid the rapidly growing population, the non-farm informal sector has become a major source of livelihood in both urban and rural areas^13^. In the future, the informal sector is anticipated to absorb the bulk of the labour force in non-farm industries^13^; hence, improving the productivity of the sector is critical for employment, income growth, and poverty reduction. While electricity access can help improve productivity of household enterprises, a growing demand for electricity also improves the financial viability and economic sustainability of electrification projects^9^.

Closing the electricity access gap requires not only an adequate amount of investment, but also a clear understanding of the requirements, technologies, risks and opportunities, preferably backed by robust data, scenarios and model projections. In this context, we aim to explore the future development of farm-related and non-farm household enterprises in SSA, their potential electricity demand, and the impact of this additional demand on energy planning in the region. In this paper we introduce a methodology to estimate PUE for farm-related and non-farm household enterprises in SSA. PUE demand for farm-related enterprises is calculated based on land use maps of current crop types, the processing operations that can take place at household level, and representative electricity requirements for each of such processes. For non-farm enterprises, we propose four statistical models and estimate them on the World Bank Enterprise Surveys: an SME uptake model, a SME electricity connection model, an SME sales model, and an SME electricity consumption model. We then use those models to predict potential SME electricity consumption across sub-Saharan Africa.

## Results

### Enterprise uptake, electricity access, annual sales, and electricity consumption

In our analysis, we present two categories of household enterprises: non-farm and farm-related. The distinction is important as farm-related businesses are seasonal and operated mostly in rural settlements. On the other hand, non-farm household enterprises operate both in rural and urban areas and while it could be seasonal for rural areas, in urban areas they operate all year. To test different possibilities, we explore a high- and low-uptake scenarios for both categories of enterprises where we cap the uptake at 50% for low-uptake scenario while high-uptake scenario is not capped.

**Figure 1:**
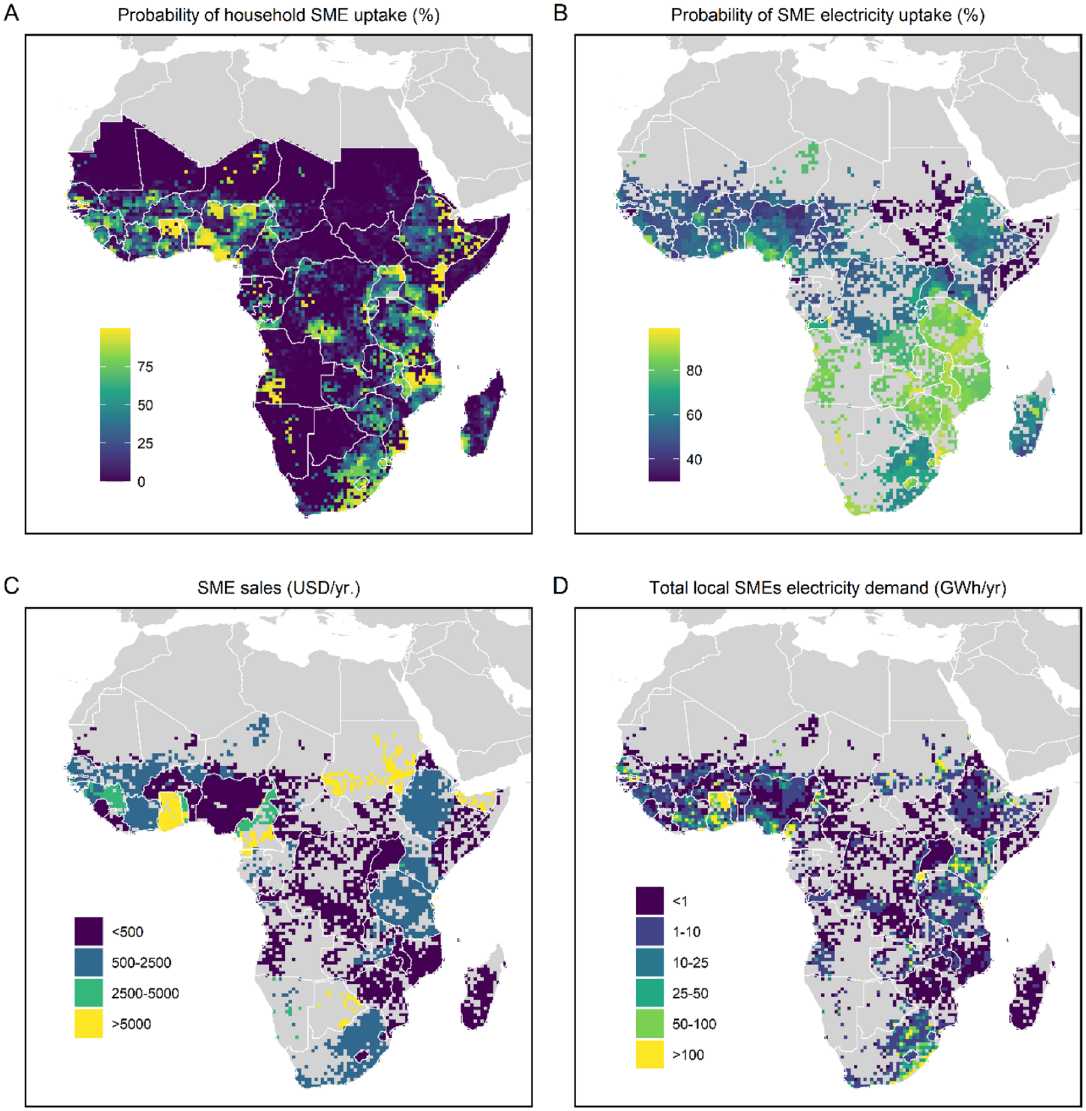
Projected growth of non-farm household enterprises. The figure presents the level of enterprise uptake, electricity connection, annual sales and electricity demand in 2030.

Figure 1 presents projected non-farm household enterprise uptake, electricity connection, annual sales and annual electricity demand in SSA in 2030. Our model projects a strong uptake of non-farm household enterprises in densely populated areas of western, eastern and southern Africa (particularly Mozambique, Zimbabwe, Tanzania, and parts of South Africa). The reason for this is that, in these regions, there is abundant supply of labour, denser markets, and, most of these countries have a high ranking of the ease of doing business. Education level of the household-head, household size, and the level of access to electricity also play a role in determining household enterprise uptake in a settlement. Vulnerability for droughts and floods has a significant negative impact on the enterprise uptake, and urban households have a higher probability of starting an enterprise compared to their rural counterparts.

Not all household enterprises gain access to electricity. Higher wealth of a settlement has a significant positive impact on electricity access of household enterprises, probably because it serves as an indicator of the ability to pay. Also framework conditions such as vulnerability to natural disaster play a significant role in gaining access to electricity, especially for households engaged in agriculture, as it limits the financial resources of the households. Given that a settlement has access to electricity, the probability of a household enterprise within this settlement to gain electricity is determined by household and settlement characteristics. Household-head literacy level, enterprise performance, and proximity to high density settlements has a positive impact on enterprise access to electricity. A positive correlation is found for mobile phone ownership, an indicator of appliance ownership and wealth of the household. Moreover, mobile phone ownership can motivate electrification by providing access to market information that can unlock knowledge and resources for productive uses of electricity. Southern African countries, with relatively high electricity access rate and high GDP, show a high level of access to electricity for household enterprises reaching up to 90%, while countries like South Sudan and Somalia show very low level of access.

The third regression model projects the enterprise sales. The annual sales of a household enterprise is determined by various factors, including enterprise characteristics, market access, access to finance, and framework conditions such as illiteracy levels. The model output shows that the lending interest rate that is used as a proxy for measuring access to finance and the probability of the enterprise having access to electricity are the strongest determinants of annual sales. Most SSA countries have high interest rates, suggesting limited availability of finance as lending is less affordable. This can inhibit enterprise operations and performance. Monthly power outages, wealth level, and illiteracy level of the settlement also show strong correlation with annual sales. The illiteracy level of a region has a negative impact on sales, as low education levels are generally associated with limited capacity to generate income, negatively affecting both household enterprise wealth and purchasing ability of consumers. This is reflected in the projected low annual sales of enterprises in Central African Republic, South Sudan and Somalia. Settlements close to densely populated areas in western, eastern and southern Africa have high annual sales, while low populated settlements in central and eastern Africa show low level of sales.

Access to electricity, annual sales, frequency of outages, and framework conditions, such as ease of doing business, and—if electricity access is currently lacking—ease of getting electricity access are the main determinants of the electricity consumption model. In the model outputs, annual sales show a strong positive correlation with enterprise electricity consumption. Parts of Ethiopia and Kenya, parts of Tanzania, eastern part of South Africa, and large parts of western Africa show high consumption levels in line with their sales. As can be expected, the frequency of outages, a proxy of the quality of electricity supply, shows a negative correlation. The number of days it takes for starting a business in a region, a proxy measure of ease of doing business, shows a positive correlation with electricity consumption. The fewer the days for starting a business, the more favourable the business environment. Hence, the household enterprises perform better, enabling the expansion of business operations and increasing electricity consumption. Similarly, ease of getting electricity access correlates positively with electricity consumption, as lower connection cost and less administrative burden favour enterprise electricity consumption.

**Figure 2:**
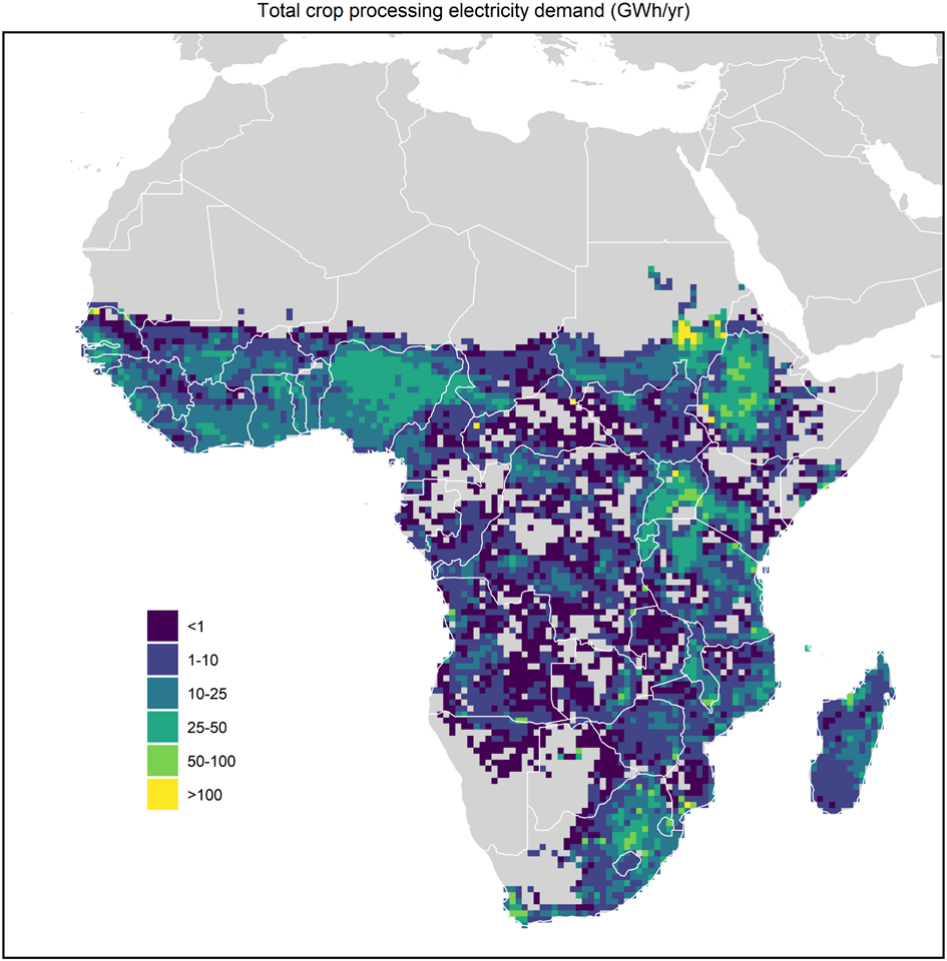
Projected growth of farm-related household enterprise. The figure presents the annual electricity demand for crop processing in SSA in 2030.

With agriculture accounting for the majority of economic activity in SSA, household enterprises focused on increased and improved crop processing are especially expected to become more relevant. Because of the positive benefits of local crop-processing, it is expected that local crop-processing operations will increase in rural areas leading to additional electricity demand. Figure 2 presents projected electricity demand of farm-related household enterprises in SSA. The electricity consumption is concentrated in high production areas in Western, Eastern and Southern Africa. This is directly related to the projected level of crop production in these areas (see Fig. 7) and the associated electricity demand of certain processes in farm-related household enterprise presented in Fig. 8. The high crop production areas in Nigeria, North-West Ethiopia, large parts of Tanzania and Mozambique, parts of South Africa, and Madagascar have a high electricity demand with the uptake of farm-related household enterprises.

### Electricity demand

**Figure 3:**
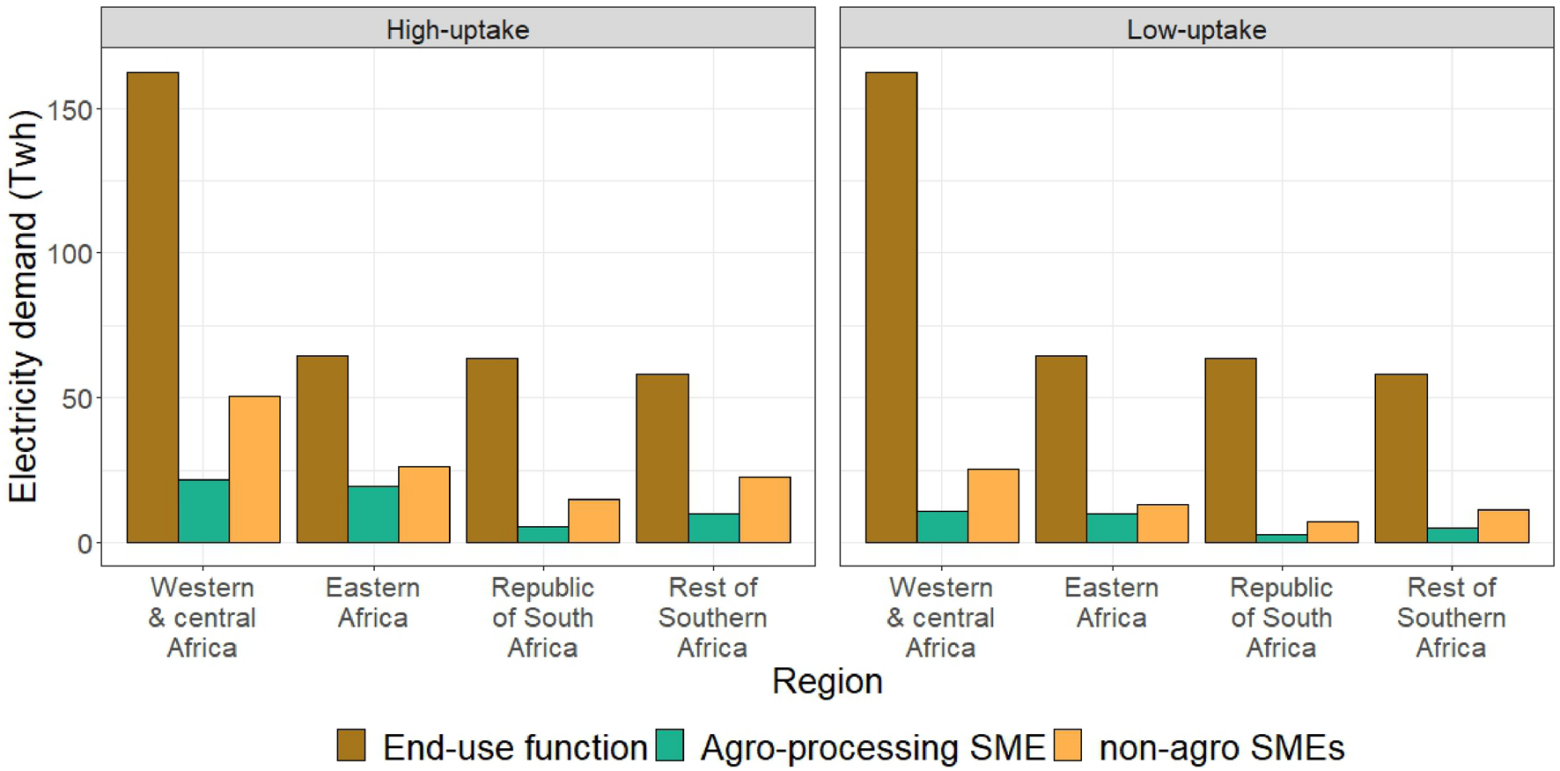
Projected electricity demand for household end-use services and productive uses in high- and low-uptake scenario in 2030.

Projected household electricity demand for residential end-use services varies considerably between regions and rural and urban settlements, ranging from 180 kWh per household per year in rural Eastern Africa to 3500 kWh in urban areas of the Republic of South Africa in 2030. Household electricity demand is projected for each end-use function, driven by factors such as household size, floor space, appliance ownership, appliance efficiency, weather, and electricity price^14^. Demand shows a considerable growth for both end-use services and productive uses. The total residential electricity demand in SSA for residential end-use services is projected at 350 TWh under universal access condition in 2030. We project that additional demand for productive uses of energy could increase this by up to 45% in high-uptake scenario (nearly 25% in low uptake scenario) (Fig. 3).

The total electricity demand for farm-related household enterprises in SSA is projected to reach 30–60 TWh in 2030. The largest share of this demand comes from west and central Africa (up to 40%) while the Republic of South Africa has the lowest share (up to 9%) of all the regions. This is driven by the large agriculture produce in western and central Africa which is more than two and half times bigger than the second biggest producing region (eastern Africa). Nigeria (the biggest economy in western and central Africa) and South Africa (a region by its own in our model) are the countries with the largest agricultural area in SSA.

Electricity demand for non-farm household enterprises is determined by the number of enterprises (which is constrained by the number of households) and electricity demand of the average enterprise in the settlement (which is related to electricity access and annual sales). Given the high number of households in western and central Africa, electricity demand for these enterprises is the highest in the region, followed by eastern Africa, the rest of southern Africa and the Republic of South Africa. The total electricity demand for non-farm household enterprises in 2030 is projected to be 60–100 TWh, equivalent to three times the current total electricity consumption of Nigeria in high-uptake scenario.

The combined electricity demand for household end-use services and productive uses of energy in 2030 in SSA ranges 430–500 TWh, an increase of 25 and 45% in the low- and high-uptake scenario, respectively, relative to the projection without PUE. Eastern Africa shows the highest increase in PUE demand relative to demand for end-use services (15–30% for farm and 20–32% for non-farm enterprises). This is the reflection of the fact that household electricity demand in the region is very low and, in a region where 44% of citizens are living on less than $1.90 per day, households are pushed into starting businesses trying to avoid slipping deeper into poverty. South Africa has the lowest relative increase of all regions (16–30%) as household electricity consumption in the country is already the highest in SSA.

### Electrification technology

**Figure 4:**
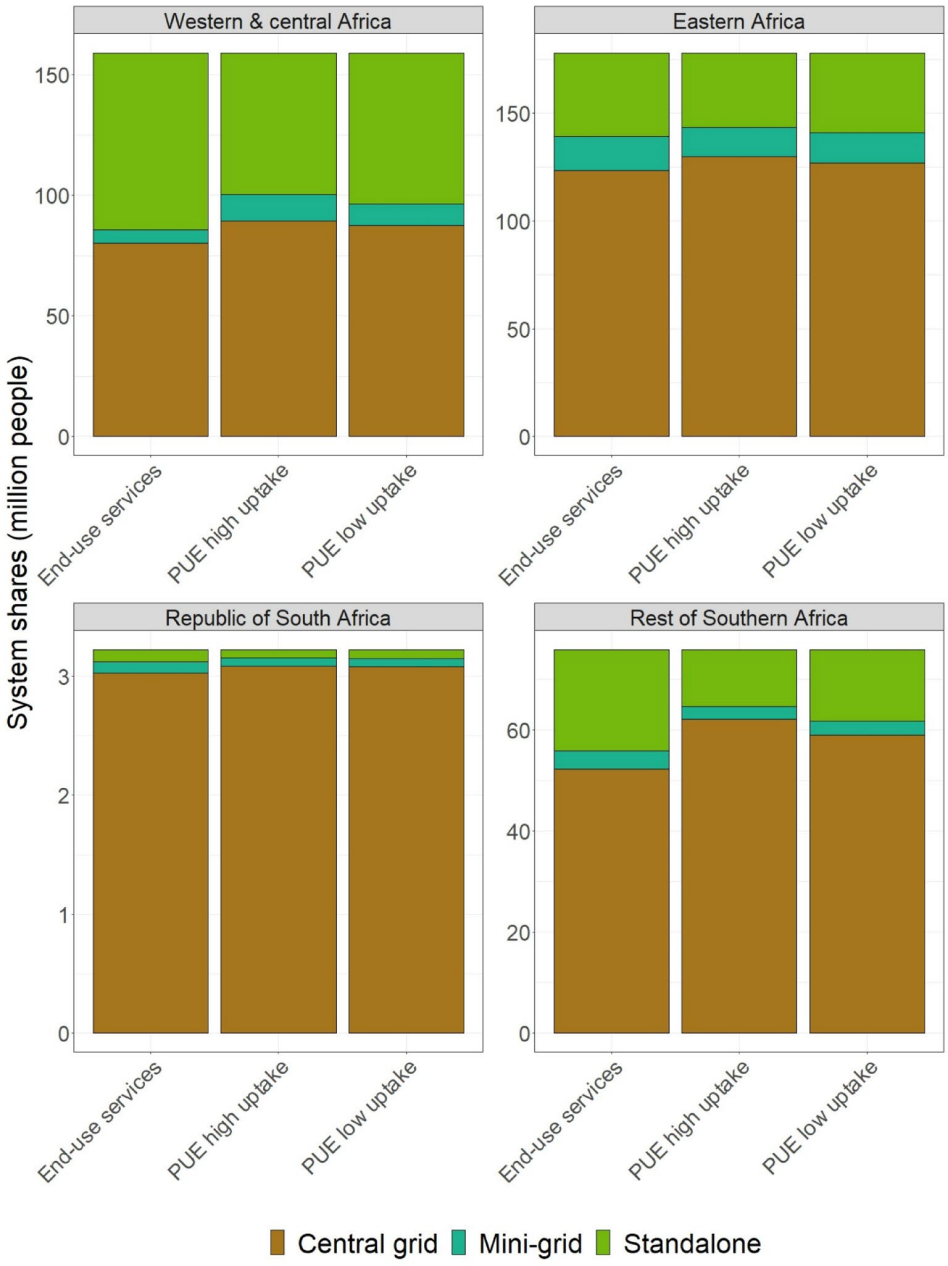
Electrification system shares on top of the baseline access with and without accounting for PUE.

The mix of electrification technologies is largely determined by the relative cost of the technologies. In the baseline, where only 65% of the population is projected to have access to electricity, the central grid plays the main role. Providing additional access requires additional generation capacity in the central grid, which mostly means scaling up of the existing generation capacity within regions, expansion of transmission and distribution lines, and a few off-grid systems. The model considers diesel, solar, wind, hydro, and two hybrid mini-grid systems and solar and diesel standalone systems as options for electrification in addition to the central grid. Under universal access without PUE, 260 million more people get access through the central grid relative to the baseline (Fig. 4). Additional 25 million and 130 million people are connected to mini-grid and standalone systems, respectively. When accounting for electricity demand for productive activities, the number of people connected to the central grid increases by 20- 25 million, compared to the scenario without PUE. The number of people connected to mini-grid systems increase by 1–2 million, while standalone system shares decline by 20–27 million people. Most of high-consuming productive activities take place in areas that are connected to the central grid due to the characteristics of the pre-conditions for starting businesses that favour towns and cities. The modest shift between electrification systems is mainly driven by the economies of scale that improved the viability of the central grid and mini-grid systems. The share of renewables declines slightly due to the dominance of the central grid that is largely supplied by fossil-fired power plants.

### Investment requirements

**Figure 5:**
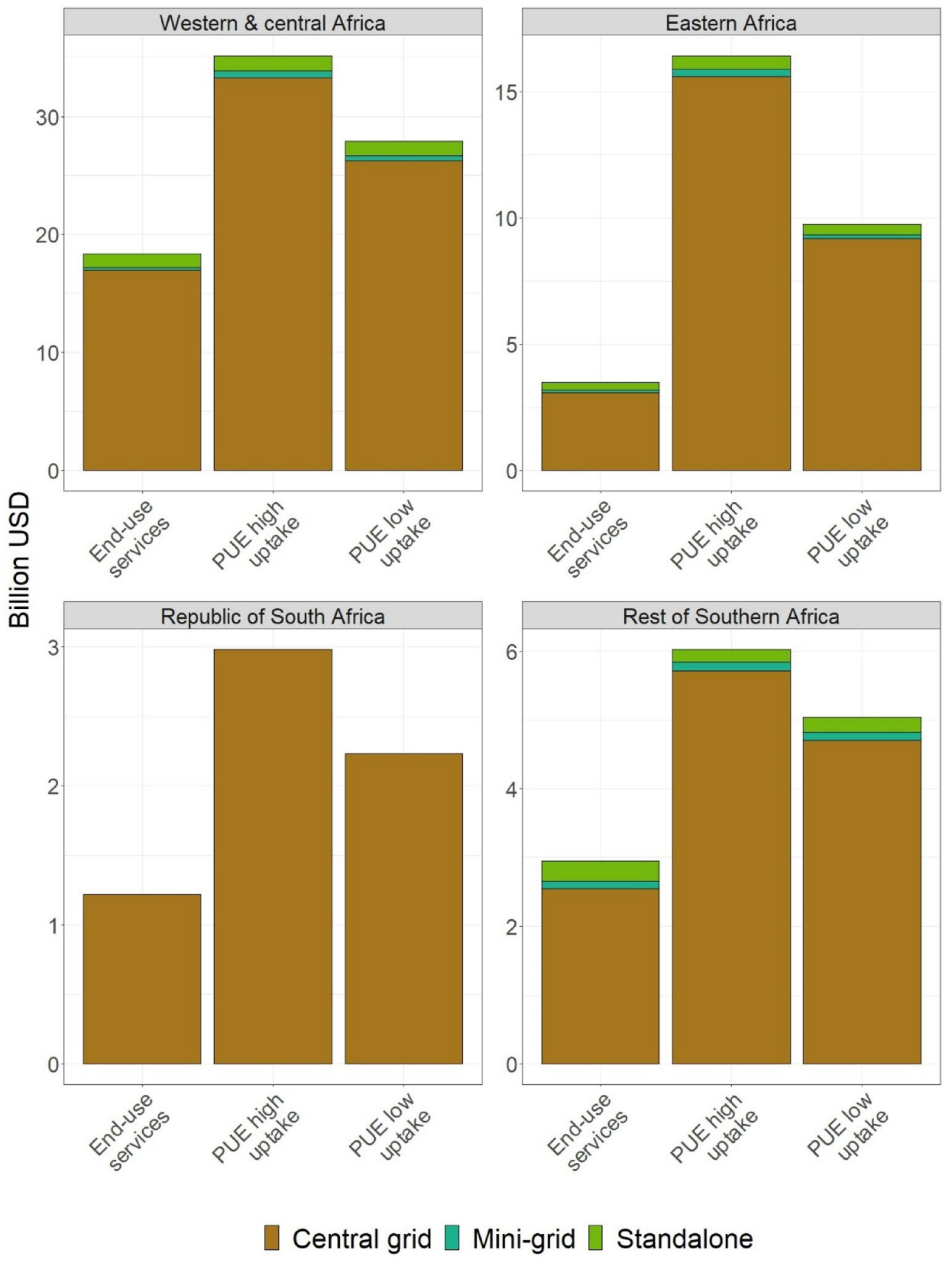
Electrification investment requirement on top of baseline investments with and without accounting for PUE.

Universal access requires increased investment in generation, transmission, and distribution systems. The average annual investment under universal access without PUE is projected to be close to 27 billion USD until 2030 on top of the baseline investment (Fig. 5). The baseline estimate is higher than what was reported by Dagnachew^15^ and IEA^16^, as there has been a very low level of investment in the energy sector in the past years. Providing electricity for productive activities requires a further expansion of the generation, transmission, and distribution infrastructure. Projected investments for universal access to electricity in SSA reaches 45–60 billion USD a year with PUE, on top of the baseline investment. Most of the investment is for expanding the transmission and distribution network and (additional) generation capacity for central-grid and mini-grid systems. This investment requirement does not include investments for the acquisition of appliances and equipment for productive uses. Expansion of the electricity system to cater demand for PUE accounts for 40% of the total electricity related investment in the residential sector of SSA.

## Discussion and conclusions

The variations in the electrical load due to a growing demand for household-level productive uses of electricity affects the sizing and operation of electricity systems. Underestimating the demand could cause supply shortages and power outages, while, on the other hand, overestimating it could lead to high investment in generation capacity and, eventually, higher electricity prices. Despite the dominance of informal employment in SSA, there is little empirical evidence on the emergence, performance and energy requirements of micro and small enterprises in the region. This paper addresses this gap by developing a methodology to model the trajectory of enterprise uptake, performance, and electricity consumption, albeit simplified assumptions. This paper presented a methodology (1) to calculate the electricity demand of farm HH enterprises, (2) to project the uptake and performance of non-farm HH enterprises, and (3) to estimate the impact of the combined demand for PUE on the electricity system in SSA. The main purpose of the paper is to explore the impact of demand for PUE on the electrification technology choices and the required investment for universal access to electricity in SSA. PUE leads to a 45% increase in total residential electricity demand and a 70% increase in electricity investments in 2030 relative to universal access without PUE.

The main challenge was identifying the right balance of understanding bottom-up factors and translating these into variables that are available SSA-wide and represent a general picture for a heterogeneous group of microenterprises. Despite the limitations in availability and accessibility of relevant data, these study developed models which deliver reasonable projections for the short and medium term. Different modelling options were tested for their feasibility and quality to identify the modelling options that best fits the objective. The long list of variables identified and tested can be found in Appendix 4.

Harnessing the income generation potential of electricity is not straightforward. Access to electricity is a necessary but not sufficient condition for income generation and poverty reduction. Other important factors, such as access to electric appliances, access to markets, access to finance, entrepreneurial skills, access to other critical infrastructure and services, the quality and reliability of the electricity supplied, acceptance and understanding of the uses of electricity and electric appliances, and affordability of electricity play an important role^17^. Besides, there are several factors which could lead to an increase in business growth. As a lot of farming in SSA is subsistence farming, there is potential that households begin to focus more on one crop or one product and begin to trade within their community than to depend on own production of various goods. Furthermore, local population growth and improved access to adjacent markets by improved infrastructure could lead to market growth. However, an in-depth assessment of these factors is beyond the scope of this paper.

Our analysis shows the general impact of attributing insufficient weight to the role of PUE in electricity planning could have on the electricity system operation. An additional important consideration is that household electricity use is most likely to be concentrated in the morning and evening hours, while relatively high-consuming PUE activities are likely to concentrate during daytime. Besides, some productive use activities show periodic fluctuation as production is seasonal^8^. Therefore, to understand the impact of PUE on system sizing and the cost of electrification projects in greater detail, it is important to study the hourly variation of electricity demand. The study also does not address the role of accelerated energy efficiency in reducing electricity demand in households.

The low level of electricity consumption is frequently mentioned as one of the barriers for electrification projects in rural parts of SSA^18^. Introducing the concept of PUE in electricity system planning changes the landscape considerably. PUE stimulates the creation of wealth by creating or expanding businesses, increasing incomes, and capturing value within communities that drives the growth of activities further. Stimulating PUE can increase the viability and sustainability of the investment and support further improvement of electricity access. Nonetheless, for PUE to have an impact, households should also be provided with information and resources about how and where to acquire appliances and equipment for their household enterprises. Similarly, access to financing options to acquire the required equipment is essential. The priority should lie with sourcing these appliances and equipment locally to stimulate economic activities, as well as, to ensure the availability of assistance for acquiring, installing, operating, and maintaining their equipment. By increasing the consumption of electricity in rural parts of SSA as a result of the local economic development, PUE improves the financial feasibility of off-grid electrification systems. To achieve all these aims, it is crucial to ensure private investors are willing to invest under given national investment conditions^19^, and smart business models that reduce risk and upfront investment barriers are widely adopted^20,21^.

This paper presented a methodology to project electricity demand for productive uses in household enterprises in SSA and show that taking PUE into electricity demand forecasting can have a considerable impact on sizing the system capacity, investment requirement, and emissions. Based on the model output, we present the following conclusions.

*Growing productive activities increase household electricity demand significantly and systems should be built to accommodate this increase.* Facilitating the uptake and sustainability of households’ farm-related and non-farm productive activities is crucial for socio-economic development. Access to electricity increases the efficiency of some of these activities, increases productivity, reduces transport cost, and relieves individuals from this time and labour-intensive activity. PUE could increase household electricity demand in SSA by 45% in 2030 compared to the universal access to electricity without productive activities. Since this will not happen overnight, decentralized systems should be built ready for integration to a larger electricity network to accommodate the large demand generated by productive activities.

*PUE could have a significant positive impact on the operational efficiency and financial viability of off-grid systems*. Micro, Small and Medium Enterprises play an important role in SSA’s economy, hence, active facilitation of PUE provides an opportunity to stimulate income generating activities for farmers and their communities, and non-farm enterprises. The ability to project electricity demand for productive uses is important for the economic feasibility and optimal operation of electrification systems. From the projected household electricity demand in SSA in 2030, over 15% of the increase stems from farm-related productive activities and nearly 30% from non-farm household enterprises. Most rural communities depend on seasonal income and PUE empowers end-user capacity and willingness to pay by generating additional income and lowering the cost of supply by taking advantage of economies of scale. Income from PUE can be used to cross-subsidize the cost of electricity to the poorest segment of society to facilitate access.

*PUE requires an increased level of investment in the electricity system to accommodate growing economic activities in households*. Increasing access to PUE could be an important catalyst for unlocking jobs, creating incomes and delivering social impacts in local communities in SSA. However, this comes at high upfront cost as expanding electricity access including for PUE requires a significant level of increase in baseline level of investment, particularly for central grid based generation, transmission and distribution. From the total investment for universal access to electricity, 70% is the result of the increased demand for productive electricity uses. In a region where public finance is constrained, there is urgent need to mobilize private finance for power sector investment by reducing the (perceived) risk on the regulatory, technological, political and market development of the energy market.

## Materials and methods

In this section we present the research methodology, the data used and the data sources. Demand for productive uses of electricity is determined for two types of household enterprises; non-farm and farm-related. The research developed a methodology to project the electricity consumption by these enterprises using the IMAGE modelling framework^22^ that includes a purpose designed bottom-up household electrification module^4^.

### Non-farm household enterprises

Research on uptake and growth of Micro, Small and Medium Enterprises (MSMEs) in SSA is limited and since the informal economy cannot be directly observed in official statistics, its magnitude needs to be estimated. Hence, we chose an explorative research method and developed four statistical models based on household survey microdata from Nigeria and enterprise survey data from thirteen countries (Nigeria, The Gambia, Namibia, Swaziland, Uganda, Burkina Faso, Cameroon, Cape Verde, The Democratic Republic of the Congo, Rwanda, Mozambique, Ivory Coast, Kenya and Zimbabwe). These models are: a binomial logit regression on the probability of a household to be engaged in entrepreneurial activity (“uptake model”), a binomial logit regression on the likelihood of household enterprises to get access to electricity (“connection model”), a multiple linear regression model on the total annual sales of an enterprise (“sales model”), which was found to be a significant determinant for the last model, a multiple linear regression on the electricity consumption of an enterprise (“consumption model”), discussed in greater detail below. Note that the models are estimated with a predictive goal, rather than an inferential one. Thus, the estimated coefficients bear no causal interpretation. Rather, the predictions of these four models are soft-linked into the IMAGE-TIMER modelling framework. The results of the regression analysis are presented in the supplementary material.

A literature review gave insights on important predictors on enterprise uptake, sales and electricity consumption that could serve as independent variables (see Appendix 3 for an overview of the sources). These variables are mostly socio-demographic characteristics of households and individuals, enterprise and entrepreneur characteristics, indicators of market access including access to infrastructure and relevant resources, and several other framework conditions especially regarding socio-political and development factors. We used either georeferenced data on household-level entrepreneurship, if available, or data on variables which could approximate other determinants (e.g. the share of population with access to radios was tested as an indicator of access to information). Besides the variables from IMAGE (such as the number of households), the main sources of variables were the World Bank Household and Enterprise Surveys, the World Bank Doing Business Indicators, the World Bank Development Indicators and the Demographic and Health Survey of the USAID (see Appendix 3 for sources). The variables were tested in different combinations to find the functions with the best predictive power that would also meet certain general quality criteria such as plausibility, significance of variables below the 0.05 statistical significance level, sufficient correlation between the dependent and independent variables, sufficient sample size, normal distribution of residuals, low multicollinearity (VIF < 10 for non-dummy variables) and low heteroskedasticity.

First, the propensity of households to engage in entrepreneurial activities is assessed. The logistic regression function for entrepreneurial activity of a household is given by:

1 $$l_{U} = log\left( {\frac{{P_{i} }}{{1 - P_{i} }}} \right) = \beta_{0} + \mathop \sum \limits_{j} \beta_{j} X_{i} + \mathop \sum \limits_{k} \beta_{k} D_{k}$$

where $l_{U}$ is the logit term of the probability $P_{i}$ that a given household ($i$) will operate an enterprise, $X_{i}$ is a continuous variable, $D_{k}$ stands for a dummy variable of a categorical variable k, $\beta_{j}$ and $\beta_{k}$ are the respective coefficients of the variables. $\beta_{0}$ is a constant term or intercept. The logistic regression equation can be used to calculate the probability of the households in a grid cell to pick up a business by inserting the according data for each grid cell and solve the equation for $P_{i}$.

2 $$P_{i} = \frac{{e^{{l_{U} }} }}{{e^{{l_{U} }} + 1}}$$

As data is given on a grid cell rather than on a household level, the household $i$ represents the average of all households in its grid cell ($g$).

3 $$P_{i,g} = P_{1,g} = P_{2,g} = P_{3,g}$$

Applying the law of large numbers, it will be assumed that the probability of uptake for household $i$ in a grid cell is equal to the share of HH with EP for that grid cell, i.e. it will give the number of households with EP ($hh_{ep}$) per total number of HH ($hh_{total}$).

4 $$P_{i} \; = \;\frac{{hh_{[ep,g\}} }}{{hh_{total,g} }}$$

Furthermore, the number of households with an enterprise is assumed to be equal to the number of enterprises, even though in reality some households might have several enterprises.

5 $$hh_{ep,g} = ep_{g}$$

The calculated probability obtained from the logit provided by the regression function can therefore be multiplied with the number of households given by IMAGE to model the number of enterprises in a grid cell.

6 $$ep_{total, g} = hh_{total,g} *P_{i}$$

Second, the probability of entrepreneurial activities to have electricity is studied. Since not all enterprises can be expected to get an electrical connection, the next step in the projection was to identify what factors determine whether an enterprise has an electrical connection or not. The model was again based on a logistic regression

7 $$l_{C} = log\left( {\frac{{Q_{m} }}{{1 - Q_{m} }}} \right) = \beta_{0} + \mathop \sum \limits_{j} \beta_{j} X_{i} + \mathop \sum \limits_{k} \beta_{k} D_{k}$$

where $l_{C}$ is the logit term of the probability $Q_{m}$ that a given EP ($m$) has an electrical connection. Again, the according data can be inserted, and the equation can be solved for the probability of an EP to have an electrical connection:

8 $$Q_{m} = \frac{{e^{{l_{C} }} }}{{e^{{l_{C} }} + 1}}$$

As the data is given on grid level again instead of enterprise-level and EP $m$ stands for all enterprises in a grid cell $g$, it is assumed that the probability of connecting of the average enterprise assumed by the model will be equal to the share of enterprises with a connection ($ep_{c}$) as part of the total number of enterprises ($ep_{total}$) in a grid cell.

9 $$Q_{m,g} = Q_{1,g} = Q_{2,g} = Q_{3,g}$$

10 $$Q_{m} \; = \;\frac{{ep_{[c,g\}} }}{{ep_{total,g} }}$$

Therefore, the number of enterprises with electrical connection can be calculated by multiplying $Q_{m}$ with the total number of enterprises in a grid cell.

11 $$ep_{c, g} = ep_{total,g} *Q_{m}$$

Third, the volume of sales of those entrepreneurial activities is statistically analysed. As the level of sales is an essential predictor for electricity consumption, the first step was to build a linear regression model for the sales of an enterprise. In this case, the World Bank EP surveys were used as a sample to test different variables for their ability to predict the annual sales of an enterprise. The linear regression function is given by

12 $$s_{m} = \beta_{0} + \mathop \sum \limits_{j} \beta_{j} X_{i} + \mathop \sum \limits_{k} \beta_{k} D_{k} + \varepsilon_{j}$$

where $s_{m}$ is the value of the dependent variable, here the total annual sales in USD of HH enterprise $m$. Inserting the data for each grid cell gives the expected sales of each enterprise in the grid cell. The calculated sales can then be inserted into the connection logistic regression function and the linear electrical consumption regression function.

Finally, the electricity consumption is modelled with a multiple linear regression function using the EP Surveys as database and selected predictor variables as well as the previously computed sales:

13 $$el_{m} = \beta_{0} + \mathop \sum \limits_{j} \beta_{j} X_{i} + \mathop \sum \limits_{k} \beta_{k} D_{k} + \varepsilon_{j}$$

where $el_{m}$ is the value of the dependent variable, here the annual electricity consumption in kWh, of an EP $m$. The values for electricity consumption can then be multiplied with the number of connected EP in the grid cell to calculate the total expected electricity consumption in kWh per grid cell.

14 $$el_{g} = el_{m} *ep_{c,g}$$

**Figure 6:**
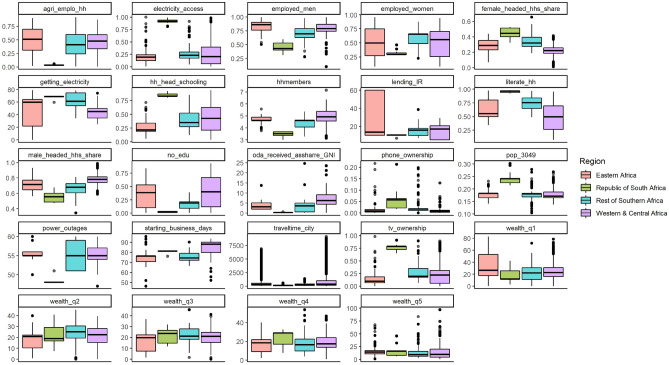
Input data for regression analysis. The figure shows some of the input data used in the regression analysis. The data is given at a gridcell level (0.5° × 0.5° cell). In the TIMER model, SSA is divided into more than 8000 grid cells.

Figure 6 presents a selection of the input data used for the regression analysis and the results of the regression analysis are presented in the Appendix 1.

### Farm-related household enterprises

**Figure 7:**
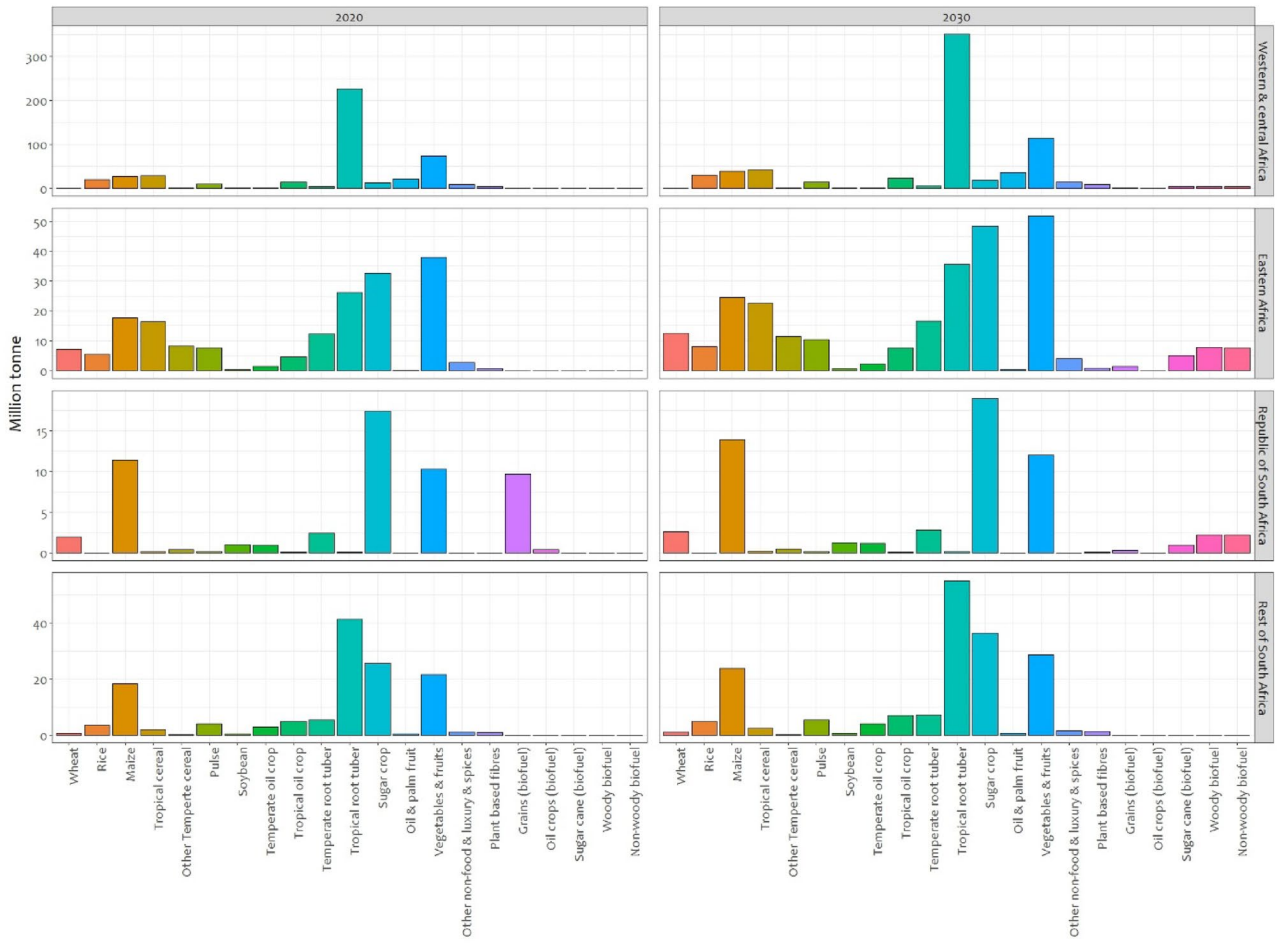
Regional distribution of the common types of crops produce in 2020, 2030, and 2050. This figure presents the historical and projected total production of common regional crops in million tonnes in Western and central Africa, Eastern Africa, Republic of South Africa, and the rest of southern Africa. The projected production is based on SSP2, middle-of-the-road baseline projections.

The research on farm-related household enterprises involved in crop processing consisted, to large extent, of an extensive literature review of both academic and grey literature (see Appendix 2 for an overview of the sources). The goal was to find out which common products and processing operations exist for the most important crops in SSA and what the respective electricity needs are. In turn, IMAGE-Land^23^, the land module of the IMAGE integrated assessment model, provides information on common crop types in the regions and the potential yield in the short and medium term (see Fig. 7). Table 1 shows the selected crops for this study based on their presence across the entire region of SSA. The “Crop category” column shows the crops given in IMAGE, the column “Crops represented” is the explanation of what is summarized in the respective categories.

**Table 1:** Common types of crops grown in SSA.

Crop category	Crops represented
Wheat (spring/winter)	Temperate cereals (wheat, rye, oats, barley, triticale)
Rice	Rice
Maize	Maize
Millet	Tropical cereals (millet, sorghum)
Field peas	Pulses
Sugar beet	Temperate roots and tubers
Cassava	Tropical roots and tubers
Sunflower	Sunflower
Soybean	Soybean
Groundnut	Groundnut
Rapeseed	Rapeseed
Sugar cane	Sugar cane

**Figure 8:**
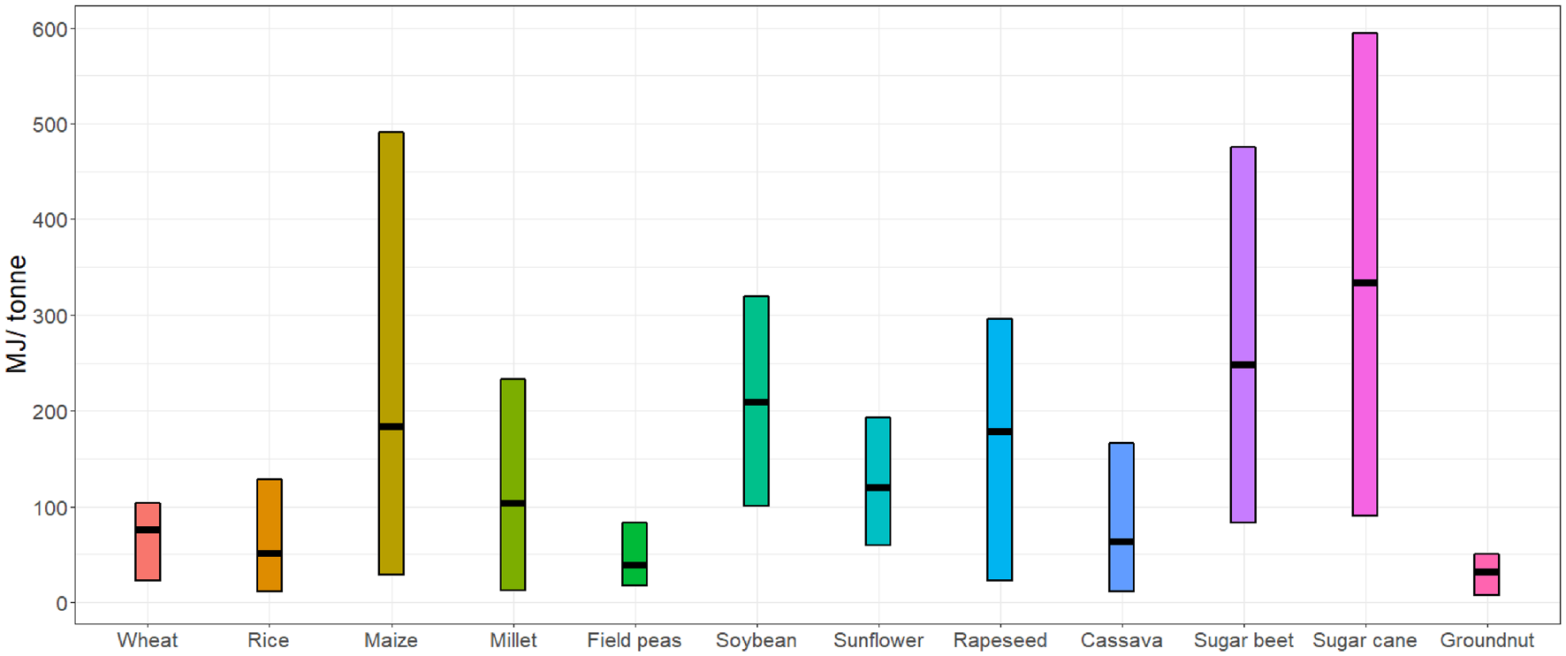
Electricity demand per processing of a specific crop in kwh/kg. The figure shows the range of electricity demand for processing in a HH enterprise based on an extensive literature screening, the black line shows the mean value used in the model.

After identifying the common crop types in SSA, the next step was to identify all the operations involved in processing these crops to make the most common products and identify the ones that can take place at household enterprises. Once these processing operations were identified, the electricity demand (kWh/kg) for each operation per unit of crop yield was determined based on literature review (see Fig. 8). The total electricity required for crop processing at a grid-cell is calculated by:

15 $$E_{agro,g} = \mathop \sum \limits_{c}^{1} P_{c,g} * e_{c}$$

where, $E_{agro,g}$ is the electricity required for agro processing in a grid-cell, $P_{c,g}$ is the annual total produce of crop c, and $e_{c}$ is the electricity required for processing crop c at a HH enterprise.

### The IMAGE model

The main instrument we use to project electricity access, electricity demand and related emissions and investments is the IMAGE model. IMAGE is an Integrated Assessment Model, a useful tool to explore the interaction between society, the biosphere and the climate system to assess sustainability issues such as climate change and biodiversity. The model framework includes a detailed description of the energy and land-use system and simulates most of the socioeconomic parameters for 26 regions. SSA is divided in four regions in IMAGE: Western and central Africa, Eastern Africa, the Republic of South Africa, and the rest of Southern Africa. Important inputs to the model are future developments of population, the economy, lifestyles, policies, and technology change. The strength of the IMAGE modelling framework is that it allows looking at the various aspects related to the transition toward universal and sustainable access to modern energy in an integrated way, including modern energy demand and supply, the availability of traditional biomass, and greenhouse gas emissions related to household energy consumption. The IMAGE model framework encompasses the TIMER energy-system simulation model^24^ and the LPJmL model, which is a global dynamic vegetation, agriculture and water balance model^25^.

TIMER is part of the IMAGE model that is designed to allow modelling long-term trends in energy consumption and generation and associated greenhouse gas (GHG) emissions^4,14,26^. The tool is used to model a bottom-up least cost electrification pathway as well as the implications of different policy scenarios on the regional electricity system. It can be used to make projections about future electricity access rates, choice of electrification technologies and associated investment needs either under given scenario assumptions or for achieving specific electrification targets. The household electrification component of the model describes household energy demand and the fuel mix for five income classes, for both rural and urban households. The model is based on a temporal unit of years and a spatial resolution of 0.5° × 0.5° grid cells with information on cost of power generation technologies, population density, household electricity demand, cost of transmission and distribution, technical potentials of renewable energy sources, and distance from existing power lines^4^.

The LPJmL model is a global vegetation, hydrology, and crop growth model that dynamically simulates yields as represented by 12 most important crop types globally^25^. Crop productivity is computed following representation of photosynthesis, maintenance and growth respiration (similar to natural vegetation) and with additional mechanisms for phenological development, allocation of photosynthesis to crop components (leaves, roots, storage organ, mobile pool/stem), and management. The IMAGE Land-use allocation model (Component Land-use allocation) is used to determine irrigation patterns, while other management decisions—such as when to sow a crop, which crop to choose, and how much irrigation water to use—are made internally. LPJmL simulates yields per crop under optimal management intensities for each grid cell (0.5° × 0.5°) and irrigation system as well as irrigation water requirements, which is input to the IMAGE Land-use allocation model (Component Land-use allocation) for simulations of land-use change dynamics.

### Scenarios

The study presents the result of three scenarios; baseline (BL), universal access (UA), and universal access with productive uses (UA-PUE). These scenarios are based on the exogenous assumptions and projections of the SSP2 scenario of the set of Shared Socioeconomic Pathways, as implemented in IMAGE^27^. The SSPs present five distinct global socio-economic pathways that describe how important societal aspects evolve in the future that imply a range of challenges for climate change mitigation and adaptation^27,28^. The drivers of energy demand addressed in SSPs include population growth, economic development, rate of technology change, and urbanization rates. SSP2 describes a world where social, economic, and technological trends do not diverge significantly from historical patterns. The socio-economic projections for SSA and sub-regions under BASELINE are presented in Table 2.

- *BL scenario*: electrification rates are determined endogenously to the model, based on GDP per capita, population density, and urbanization rate. Electricity consumption of households considers electricity demand for end-use services, that is for space heating, space cooling, water heating, cooking, lighting and other household appliance use.
- *UA scenario*: household electricity consumption is similar to the BL scenario. However, under this scenario, all SSA households are projected to have access to electricity by 2030. Access to electricity increases linearly from the regions’ 2020 levels to a 100% in 2030.
- *UA-PUE scenario*: unlike the other two scenarios, electricity consumption of households under this scenario includes electricity consumption for productive uses by household enterprises, as estimated through our regression models. All households are projected to have access to electricity in 2030, similar to the UA scenario.

**Table 2:** Socio-economic data under SSP2 scenario^6,26,27^.

Region	Population (million)		GDP per capita (USD/cap)		Urban population (%)	
	2010	2030	2010	2030	2010	2030
Sub-Saharan Africa	864	1329	1979	3721	37%	48%
Western & central Africa	417	656	1586	3400	44%	55%
Eastern Africa	260	403	1228	2406	24%	33%
Republic of South Africa	50	59	9252	16,407	62%	72%
The rest of southern Africa	137	211	1943	3682	35%	46%

### Supplementary Information

Supplementary Information.

## Data Availability

The sources of the input data for the statistical models and socio-economic projection are presented here. Model outputs are available on request. (1) World Bank’s Enterprise Surveys: https://www.enterprisesurveys.org/en/methodology. (2) World Bank Doing Business Indicators: (i) starting a business https://www.doingbusiness.org/en/methodology/starting-a-business, (ii) getting electricity https://www.doingbusiness.org/en/methodology/getting-electricity. (3) DHS Program https://www.dhsprogram.com/What-We-Do/Methodology.cfm. (4) World development indicators: https://datacatalog.worldbank.org/dataset/world-development-indicators. (5) UNDP http://hdr.undp.org/en/content/human-development-index-hdi. (6) SSP database https://tntcat.iiasa.ac.at/SspDb/dsd?Action=htmlpage&page=10.

## Code availability

This study uses the IMAGE-TIMER model as the main instrument of the study. The complete documentation of the model can be found on https://models.pbl.nl/image/index.php/Welcome_to_IMAGE_3.2_Documentation. More details and additional code can be made available on request.
