# Supplementary material for: Energy planning in Sub-Saharan African countries needs to explicitly consider productive uses of electricity
Source: Sci Rep. 2023 Aug 10;13:13007. doi: 10.1038/s41598-023-40021-y (PMC10415313; doi:10.1038/s41598-023-40021-y)
Supplement: Supplementary file 1 — Supplementary Information. [file 41598_2023_40021_MOESM1_ESM.docx]

# Supplementary material

## Appendix 1: Regression Modelling Results

### Uptake Logistic Regression Model

Table 1: Variables in the final uptake regression function with their coefficients (B), level of significance and exponentiated coefficients (exp(B)).

|  | | **B** | **Sig.** | **Exp(B)** |
| --- | --- | --- | --- | --- |
| Constant | | -1.029 | 0.000 | 0.357 |
| HH characteristics | Number of HH members | 0.061 | 0.000 | 1.063 |
|  | Share of pop. in lowest WQ in state | 0.032 | 0.000 | 1.032 |
|  | Share of pop. in low-mid WQ in state | -0.014 | 0.000 | 0.987 |
|  | Share of pop. in middle WQ in state | 0.002 | 0.000 | 1.002 |
|  | Share of pop. in mid-hi WQ in state | 0.035 | 0.000 | 1.035 |
|  | Agricultural activity in HH | -0.156 | 0.000 | 0.856 |
|  | HH ownership of TVs | 0.030 | 0.000 | 1.030 |
|  | HH ownership of mobile phone | 0.332 | 0.000 | 1.394 |
|  | Experience of drought in community (3y) | -0.061 | 0.000 | 0.941 |
|  | Experience of flood in community (3y) | -0.032 | 0.000 | 0.969 |
| HHH characteristics | Sex of HHH | 0.110 | 0.000 | 1.116 |
|  | School attendance of HHH | 0.509 | 0.000 | 1.664 |
| Market access | Access to electricity in HH | 0.485 | 0.000 | 1.624 |
|  | Travel time to the next city | -3.12E-04 | 0.000 | 1.000 |
|  | Sector | 0.208 | 0.000 | 1.232 |
|  | Settlement type |  | 0.000 |  |
|  | water grid cell | -0.435 | 0.000 | 0.647 |
|  | very low-density rural grid cell | -0.172 | 0.000 | 0.842 |
|  | low density rural grid cell | -0.479 | 0.000 | 0.619 |
|  | rural cluster grid cell | -0.546 | 0.000 | 0.579 |
|  | suburban or peri-urban grid cell | -0.871 | 0.000 | 0.419 |
|  | Distance to nearest major road | 0.009 | 0.000 | 1.009 |
|  | Distance to nearest population centre | -2.11E-04 | 0.000 | 1.000 |
|  | Number of urban cells | 0.009 | 0.000 | 1.009 |
|  | Distance to nearest market | -0.002 | 0.000 | 0.998 |
| Framework conditions | Share of women employed in state | -0.026 | 0.000 | 0.974 |
|  | Share of men employed in state | 0.016 | 0.000 | 1.016 |
|  | Doing Business: Expected days to start a business | -0.016 | 0.000 | 0.984 |

N = 4,781

Nagelkerke R^2^ = 0.177

### Connection to Electricity Regression Model

Table 2: Variables in the final connection regression with their coefficients (B), level of significance and exponentiated coefficients (exp(B)).

|  | | **B** | **Sig.** | **Exp(B)** |
| --- | --- | --- | --- | --- |
| Constant | | -2.951 | 0.000 | 0.052 |
| HH characteristics | Agricultural activity in HH | -0.442 | 0.014 | 0.626 |
|  | HH ownership of mobile phone | 0.494 | 0.000 | 1.689 |
|  | share of HH in hi WQ in state | 0.043 | 0.000 | 1.049 |
| Entrepreneur characteristics | Literacy of manager | 0.608 | 0.000 | 1.896 |
| Enterprise characteristics | ln Sales in the last FY (USD) | 0.252 | 0.000 | 0.643 |
| Market Access | Number of urban cells | 0.043 | 0.000 | 1.043 |
| Framework conditions | Experience of drought in community (3y) | -0.784 | 0.000 | 0.438 |

N = 1,659

Nagelkerke R^2^ = 0.461

### Sales Linear Regression Model

Table 3:Variables in the final sales regression with their coefficients (B), level of significance and z-score standardized coefficients

|  |  | **B** | **Sig.** | **Standardized B** |
| --- | --- | --- | --- | --- |
| Constant | | 8.968 | 0.000 |  |
| EP characteristics | Power outages in firms in a typical month (number) | -0.049 | 0.000 | -0.333 |
|  | share of pop. in lowest WQ in state | -0.011 | 0.022 | -0.112 |
|  | share of pop. in low-mid WQ in state | -0.006 | 0.470 | -0.051 |
|  | share of pop. in middle WQ in state | -0.003 | 0.619 | -0.023 |
|  | share of pop. in mid-hi WQ in state | -0.033 | 0.000 | -0.250 |
| Market Access | National lending interest rate (%) | -0.099 | 0.000 | -0.474 |
|  | Doing Business: Getting Electricity score | 0.063 | 0.000 | 0.462 |
|  | Share of pop. without education | -0.008 | 0.000 | -0.136 |
| Framework conditions | SSA region - Central Africa | 1.240 | 0.000 | 0.415 |
|  | SSA region -West Africa | 0.759 | 0.000 | 0.251 |
|  | SSA region - Southern Africa | 0.757 | 0.000 | 0.252 |

N = 1,609

R^2^ = 0.26

### Electricity Consumption Linear Regression

Table 4: Variables in the final consumption regression with their coefficients (B), level of significance and z-score standardized coefficients

|  |  | **B** | **Sig.** | **Standardized B** |
| --- | --- | --- | --- | --- |
| Constant | | 2.837 | 0.000 |  |
| EP characteristics | ln Sales in last FY (USD) | 0.430 | 0.000 | 0.288 |
|  | share of pop. in low-mid WQ in state | -0.058 | 0.000 | -0.361 |
|  | share of pop. in middle WQ in state | 0.022 | 0.025 | 0.106 |
|  | share of pop. in mid-hi WQ in state | -0.039 | 0.000 | -0.202 |
|  | share of pop. in lowest WQ in state | 0.020 | 0.014 | 0.145 |
| Market Access | ln number of outages in a typical month | -0.173 | 0.002 | -0.099 |
| Framework conditions | Doing Business: Expected days required to start a business | -0.042 | 0.000 | -0.637 |
|  | Doing Business: Getting Electricity score | 0.047 | 0.000 | 0.244 |
|  | SSA region - Central Africa | 0.488 | 0.029 | 0.108 |
|  | SSA region -West Africa | 1.162 | 0.000 | 0.256 |
|  | SSA region - Southern Africa | 0.420 | 0.092 | 0.102 |
|  | GNI, PPP (constant 2017 international $) | 1.715E-12 | 0.000 | 0.151 |

N = 1,159

R^2^ = 0.39

## Appendix 2: Sources of the Literature Review for Agricultural Enterprises

Table 5: Crop processing findings with indication where own assumptions of the author were used for the assessment (a).

| **Source** | **Crop** | **Country/Area** | **Size** | **Product** | **Electricity demand (MJ/kg)** |
| --- | --- | --- | --- | --- | --- |
| (Olaoye et al., 2020) | Wheat | Nigeria | Micro | Flour (75%), semolina (3%), bran 22%) | 0.101 |
| (Green et al., 2019) | Wheat | Nigeria | Medium/Large (a) | Flour | 0.023 |
| (Bassey & Schmidt, 1989) | Wheat | South Africa | Small (a) | Flour | 0.104 (a) |
| (Goyal et al., 2010) | Rice | India | Medium/Large (a) | Raw milled rice, parboiled rice | 0.057 (a) |
| (Goyal & Agrawal, 2008) | Rice | India | Medium/Large (a) | Raw milled rice | 0.019 |
| (Roy et al., 2005) | Rice | India | Medium (a) | Raw milled rice, parboiled rice | 0.076 |
| (Ahiduzzaman & Islam, 2009) | Rice | Bangladesh | Medium/Large | Parboiled rice | 0.025 (a) |
| (Roomi et al., 2007) | Rice | Sri Lanka | Medium (a) | Raw milled rice, parboiled rice | 0.087 (a) |
| (Bassey & Schmidt, 1989) | Rice | South Africa | Micro/Small | Raw milled rice | 0.052 |
| (ILO et al., 1984) | Maize | Developing Countries | Micro/Small | Maize flour | 0.031 (a) |
| (Rottger, 2006) | Maize | Africa | Micro | Maize flour | 0.079 (a) |
| (Abubakar et al., 2010) | Maize | Nigeria | Micro | Maize flour | 0.491 (a) |
| (Aliu et al., 2018) | Maize | Nigeria | Large | Maize flour | 0.231 |
| (Dozier et al., 2005) | Millet | US | Medium/Large | Milled pear millet | 0.012 (a) |
| (Ambrose et al., 2017)am | Millet | India | Micro | Flour from different millet types | 0.201 (a) |
| (Balasubramanian, 2015) | Millet | India | Micro | Milled millet | 0.024 |
| (Chin et al., 2012)c | Millet | India | Micro/Small | Milled millet | 0.026 |
| (Bassey & Schmidt, 1989) | Millet | South Africa | Small/Medium | Milled millet | 0.090 |
| (Opoku & Tabil, 2006) | Field Peas | Canada | Micro | Ground field peas | 0.045 (a) |
| (Lal & Verma, 2007) | Field Peas | India | Micro/Small | Dried, split pulses | 0.030 (a) |
| (Goyal & Agrawal, 2008) | Field Peas | India | Micro/Small | Dried, split pulses | 0.032 (a) |
| (Bassey & Schmidt, 1989) | Field Peas | South Africa | Micro/Small | Dehulled cowpea | 0.083 |
| (Chouhan & Chandrakar, 2014) | Sugar Beet | India | Large (a) | Sugar | 0.101 (a) |
| (Jensen et al., 2015) | Sugar Beet | Europe | Large (a) | Sugar | 0.083 (a) |
| (Sattari et al., 2007) | Sugar Beet | Iran and world | Medium (a) | Sugar | 0.407 (a) |
| (Jekayinfa & Olajide, 2007) | Cassava | Nigeria | Small (a) | Starch | 0.011 |
| (Tran et al., 2015) | Cassava | Thailand, Vietnam, Colombia | Small/Medium | Starch | 0.047 (a) |
| (Da et al., 2013) | Cassava | Colombia and Vietnam | Medium (a) | Starch | 0.030 |
| (Yin et al., 2019) | Cassava | Africa and Asia | Large | Starch | 0.166 (a) |
| (Spinelli et al., 2013) | Sunflower | Italy | Large (a) | Oil | 0.193 |
| (Ion & Popescu, 2017) | Sunflower | unclear | Large | Oil | 0.060 |
| (Kartika et al., 2010) | Sunflower | France | Large | Oil | 0.108 |
| (Bassey & Schmidt, 1989) | Soybean | South Africa | Small | Milled soybean | 0.101 |
| (Smith et al., 2007) | Soybean | Canada | Large (a) | Oil | 0.319 |
| (Eshetu et al., 2017) | Groundnuts | Ethiopia | Small (a) | Shelled groundnuts | 0.040 |
| (Ugwuoke & Okegbile, 2014) | Groundnuts | Nigeria | Small | Shelled groundnuts | 0.007 |
| (Atiku et al., 2004) | Groundnuts | Nigeria | Small | Shelled groundnuts | 0.050 |
| (Smith et al., 2007) | Rapeseed | Canada | Large (a) | Oil | 0.217 |
| (Fore et al., 2011) | Rapeseed | US | Medium (a) | Oil | 0.023 |
| (Chen & Chen, 2011) | Rapeseed | China | Large (a) | Oil | 0.296 (a) |
| (M. Soppimath & G. Hudedmani, 2017) | Sugar Cane | India | Large | Sugar | 0.230 |
| (Pippo & Luengo, 2016) | Sugar Cane | unclear | Large | Sugar | 0.090 |
| (Sattari et al., 2007) | Sugar Cane | Iran and world | Medium (a) | Sugar | 0.509 (a) |

## Appendix 3: Sources of the Literature Review for Non-agricultural Enterprises

Table 6: Predictors with sources and indication of relevant dependent variables

| **Category** | **Predictor** | **Source** |
| --- | --- | --- |
| **Household Characteristics** | |  |
|  | Income/level of wealth/assets | ADA (2016) (U) |
|  |  | Nagler and Naudé (2018) (U) |
|  |  | Christiaensen and Demery (2018) (U) |
|  |  | Kooijman-van Dijk and Clancy (2010) (C) |
|  |  | Nkegbe et al. (2018) (U) |
|  | number of household members | Owoo and Naudé (2015) (P) |
|  |  | Nagler and Naudé, (2017) (U) |
|  |  | Christiaensen and Demery (2018) (U) |
|  | number of rooms | Nagler and Naudé, (2017) (U) |
|  | shock experience | Efobi et al. (2019) (U) |
|  |  | Nagler and Naudé, (2017) (U) |
|  |  | Christiaensen and Demery (2018) (U) |
|  | house size | Rao (2013) (U) |
|  | agricultural activity  (precipitation) | Owoo and Naudé (2015) (P) |
|  |  | Nagler and Naudé (2017) (U) |
|  |  | Christiaensen and Demery (2018) (U) |
|  | access to information  (e.g. radio ownership) | Nkegbe et al. (2018) (P) |
| **Entrepreneur Characteristics** | |  |
|  | knowledge/skills (general) | Practical Action Consulting, (2012) (P, C) |
|  |  | Wilcox et al. (2015) (P, C) |
|  |  | Cabraal et al. (2005) (P, C) |
|  |  | Naidoo and Urban, (2012) (P) |
|  |  | Pueyo et al. (2014) (C) |
|  |  | Mayer-Tasch et al. (2013) (P) |
|  | level of education (years of schooling, literacy) | Rao (2013) |
|  |  | Mayer-Tasch et al. (2013) (P) |
|  |  | ADA (2016) (U) |
|  |  | Efobi et al. (2019) (U) |
|  |  | Nkegbe et al. (2018) (U) |
|  |  | Christiaensen and Demery (2018) (U) |
|  |  | Nagler and Naudé, (2017) (U) |
|  |  | Haggblade (2007) (U) |
|  | digital and mechanical know-how | Mayer-Tasch et al. (2013) (P) |
|  |  | Blodgett (2016) (U, P, C) |
|  | social networks (religion) | Kooijman-van Dijk (2012) (P) |
|  |  | Owoo and Naudé (2015) (U, P) |
|  |  | Kooijnman-van Dijk (U) |
|  |  | Nkegbe et al. (2018) (U) |
|  |  | Practical Action Consulting, (2012) (P) |
|  | marital status | ADA (2016) (U) |
|  |  | Efobi et al. (2019) (U) |
|  |  | Akpan et al. (2014) (U) |
|  |  | Nagler and Naudé, (2017) (U) |
|  | age | Efobi et al. (2019) (U) |
|  |  | Owoo and Naudé (2015) (P) |
|  |  | Christiaensen and Demery (2018) (U) |
|  |  | Akpan et al. (2014) (U) |
|  |  | Blodgett (2016) (U, P, C) |
|  | sex | Lecoque & Wieman (2015) (C) |
|  |  | (Maleko, 2005)Maleko (2005) (P) |
|  |  | Owoo and Naudé (2015) (P) |
|  |  | Nagler and Naudé, (2017) (U, P) |
|  |  | Christiaensen and Demery (2018) (U) |
|  |  | Mapako and Prasad (2008) (U) |
|  |  | Harsdorff and Bamanyaki, (2009) (C) |
|  |  | Akpan et al. (2014) (U) |
|  |  | Mead and Liedholm (1998) (U) |
|  | awareness and acceptance of productive uses of electricity | Renner (2017) (C) |
|  |  | Mayer-Tasch et al. (2013) (C) |
|  | personal character | ADA (2016) (U, P) |
|  | level of entrepreneurial experience | Benedikter et al. (2013) (U) |
|  |  | ADA (2016) (U) |
|  |  | Lecoque & Wieman (2015) (C) |
| **Enterprise Characteristics** | |  |
|  | Wealth, size (income, assets, capital) | Mayer-Tasch et al. (2013) (C) |
|  |  | Kooijman-van Dijk and Clancy (2010) (C) |
|  |  | Mapako and Prasad (2008) (P) |
|  |  | Harsdorff and Bamanyaki (2009) (C) |
|  |  | Ekblom (2016) (P) |
|  |  | Pueyo et al. (2014) (C) |
|  |  | Banerjee et al. (2017) (C) |
|  |  | Mead and Liedholm (1998) (P) |
|  | type/industry | Mayer-Tasch et al. (2013) (C) |
|  |  | Kooijnman-van Dijk (2008) (P, C) |
|  |  | Owoo and Naudé (2015) (P) |
|  |  | Mead and Liedenholm (1998) (P) |
|  |  | Pueyo et al. (2014) (C) |
|  |  | Harsdorff and Bamanyaki, (2009) (C) |
|  | registration and formality | ADA (2016) (P) |
|  |  | Loening and Lane (2007) (P) |
|  |  | Foster et al., (2010) (P) |
|  | location of enterprise | Olawale and Garwe (2010) (P) |
|  |  | Mayer-Tasch et al. (2013) (P) |
|  | | Nagler and Naudé, (2017) (U) |
|  |  | Christiaensen and Demery (2018) (U) |
|  |  | Pueyo et al. (2014) (C) |
|  |  | Blodgett (2016) (U, P, C) |
| **Market Access: Demand** | | Kooijman-van Dijk and Clancy (2010) (P, C) |
|  |  | Harsdorff and Bamanyaki, (2009) (P) |
|  |  | Mead and Liedenholm, (1998) (U) |
|  | population density | Kooijman-van Dijk and Clancy (2010) (P, C) |
|  |  | Kooijman-van Dijk and Clancy (2010) (C) |
|  |  | Haggbladde (200/) (U) |
|  | urban/rural migration | Nagler and Naudé, (2017) (U) |
|  | local wealth and purchasing power | Practical Action Consulting, (2012) (P) |
|  | local population/market size | Mayer-Tasch et al. (2013) (P) |
|  | number of people who transit | Blodgett (2016) (U, P, C) |
|  | level of competition | Practical Action Consulting, (2012) (P) |
| **Market Access: Supply/Input factors** | | Practical Action Consulting, (2012) (P) |
|  | access to finance (presence of banks, interest rate, required collateral, required owners’ equity contribution) | Mayer-Tasch et al. (2013) (P) |
|  |  | ADA (2016) (U) |
|  |  | FRES (2014) (U) |
|  |  | Nagler and Naudé, (2017) (U) |
|  |  | Christiaensen and Demery (2018) (U) |
|  |  | Blodgett (2016) (U) |
|  |  | Fishbein et al. (2003) (U) |
|  |  | Ekblom (2016) (P) |
|  |  | Pueyo et al. (2014) (C) |
|  |  | Loening and Lane (2007) (P) |
|  |  | Olawaleand Garwe (2010) (U, P) |
|  |  | Mead and Liedenholm (1998) (U) |
|  | cost of input factors (other than electricity) | Pueyo et al. (2014) (C) |
|  |  | Blodgett (2016) (C) |
|  | presence electricity access | Rao (2013) (P, C) |
|  |  | Mayer-Tasch et al. (2013) (P) |
|  |  | Maleko (2005) (U) |
|  |  | Prasad and Dieden (2007) (U,P) |
|  |  | FRES (2013) (U) |
|  |  | Harsdorff and Bamanyaki, (2009) (U) |
|  |  | Blodgett (2016) (U) |
|  |  | Peter et al., (2011) (U) |
|  |  | Kirubi et al., (2008) (P) |
|  |  | Lenz (2017) (U) |
|  |  | Gibson and Olivia, (2015) (U) |
|  | electricity access capacity (voltage, AC/DC…) quality (number and duration of blackouts in given time; presence of risk factors for supply) | Mayer-Tasch et al. (2013) (P) |
|  |  | World Bank (2015) (C) |
|  |  | Foster et al., (2010) (P) |
|  |  | Blodgett (2016) (C) |
|  |  | Mapako and Prasad (2008) (P) |
|  |  | Moyo (2013) (P) |
|  |  | Pueyo et al. (2014) (C) |
|  |  | World Bank (2015) (C, P) |
|  |  | Gibson & Olivia, 2010) (U) |
|  | cost of electrical connection | Mayer-Tasch et al. (2013) (C) |
|  |  | Pueyo et al. (2014) (C) |
|  | cost of electricity consumption | Pueyo et al. (2014) (C) |
| **Physical Market Access and Transportation** | | Rao (2013) (P, C) |
|  |  | Wilcox et al. (2015) |
|  |  | Mayer-Tasch et al. (2013) (P) |
|  |  | Nkegbe et al. (2018) (U) |
|  |  | Haggblade (U) |
|  | physical access to input goods (e.g. appliances) and services (e.g. maintenance) | MFAN, (2014) (P) |
|  |  | Mayer-Tasch et al. (2013) (P, C) |
|  |  | Peter et al., (2011) (P, C) |
|  | level of urbanization | Rao (2013) (P, C) |
|  |  | Mead and Liedenholm (1998) (P) |
|  |  | Akpan et al. (2014) (U) |
|  | distance to urban centers | Nagler and Naudé, (2017) (U) |
|  |  | Christiaensen and Demery (2018) (U) |
|  |  | Gibson and Olivia, (2015) (U) |
|  | presence of roads and distance to roads | Kooijman-van Dijk and Clancy (2010) (C) |
|  |  | Kooijman-van Dijk and Clancy (2010) (C) |
|  |  | Loening and Lane (2007) (P) |
|  |  | Efobi et al. (2019) (U) |
|  | quality of roads | Kooijman-van Dijk (2012) (C, P) |
|  |  | Mayer-Tasch et al. (2013) (P) |
|  |  | Loening and Lane (2007) (P) |
|  |  | Gibson and Olivia, (2015) (U) |
|  | presence of regular market/commercial center | FRES (2014) (U) |
|  |  | Mayer-Tasch et al. (2013) (P) |
|  | access to daily public transportation | diaoDiao et al. (2018) (U) |
| **Governance, institutional context and development** | | ADA (2016) (U) |
|  | conductive regulatory environment and policies | Practical Action Consulting, (2012) (P) |
|  |  | ADA (2016) (U) |
|  |  | Lecoque & Wieman (2015) (C) |
|  |  | Nagler and Naudé (2018) (P) |
|  |  | Olawaleand Garwe (2010) (U, P) |
|  |  | Blodgett (2016) (U) |
|  | effort to start a business | World Bank (2018) (U, P) |
|  | level of corruption | Olawaleand Garwe (2010) (U, P) |
|  |  | ADA (2016) (U) |
|  | political relevance and activity of locality (presence of communal administration) | Mayer-Tasch et al. (2013) (P) |
|  | level of social security | Christiaensen and Demery (2018) (U) |
|  | availability of subsidies to SMEs | ADA (2016) (U) |
|  | country | Owoo and Naudé (2015) (P) |
|  | GDP | Chu et al. (2007) (U, P) |
|  | gender equality | Etim and Iwu (2018) (U) |
|  | presence of violent conflicts | Loening and Lane (2007) (P) |
|  |  | Nagler and Naudé (2018) (P) |
|  | access to water | Cabraal et al., 2005 (P) |
|  | presence of schools | Cabraal et al. (2005) (P) |
|  |  | Mayer-Tasch et al. (2013) (P) |
|  | presence of health facilities | Cabraal et al. (2005) (P) |
|  | investment climate | Aterido (2010) (U) |
|  | access to cell phone communication | Loening and Lane (2007) (P) |
| **Other** | |  |
|  | season | Loening and Lane (2007) (P) |
|  |  | Blodgett (2016) (U) |
|  | weather (droughts) | Blodgett (2016) (C) |
| **Development Program Characteristics** | | Aterido (2013) (P) |
|  | training and information provided and quality (BDS), knowledge sharing | Mayer-Tasch et al. (2013) (P, C) |
|  |  | ADA (2016) (U) |
|  |  | Lecoque & Wieman (2015) (C) |
|  |  | Tanzania National Survey Baseline report (2012) (P) |
|  |  | Blodgett (2016) (U) |
|  |  | Fishbein (2003) (U) |
|  | monitoring progress and satisfaction | Golumbeanu and Barnes (2014) (P, C) |

Table 7: Further variables and their sources

| Night-time lights | yearly average radiance (µW·sr^−1^·cm^−2^) recorded in the night-time hours | (Elvidge et al., 2017) |
| --- | --- | --- |
| Settlement type data | categories of settlement type: water grid cell, very low-density grid cell, low density rural grid cell, rural cluster grid cell, suburban or peri-urban grid cell, semi-dense urban cluster grid cell, dense urban cluster grid cell, urban centre grid cell | (Florczyk et al., 2019) |
| Urban cells | number of urban cells in the 5-km radius around the coordinates |  |
| Distance to markets | distance in km to core grid cells |  |
| Distance to the nearest urban centre (>20.000 inhabitants) | In km |  |
| Travel time to the next city (>50.000 inhabitants) | in minutes based on the fastest transportation mode, based on existing roads traffic etc. | (Weiss et al., 2018) |
| Road density | total length of roads in the 5-km radius around the coordinates | (CIESIN, 2013) |
| Distance to the next road | In km |  |
| Occurrence of droughts and floods | In past three years | (Beguería & Vicente Serrano, 2016) |

## Appendix 4: Long list of variables included in the testing phase

*Table 3. Predictors for EP uptake, performance and electricity consumption as identified in literature.*

| **Category** | **Predictor** |
| --- | --- |
| Household Characteristics | Income/level of wealth/assets (U, C) |
|  | number of hh members (U, P) |
|  | number of rooms (U) |
|  | shock experience (U) |
|  | house size (U) |
|  | agricultural activity (precipitation) (U, P) |
|  | access to information (e.g. radio ownership) (P) |
| Entrepreneur Characteristics | knowledge/skills (general) (P, C) |
|  | level of education (years of schooling, literacy) (P, U) |
|  | digital and mechanical know-how (U, P, C) |
|  | social networks (religion) (U, P) |
|  | marital status (U) |
|  | age (U, P, C) |
|  | sex (U, P, C) |
|  | awareness and acceptance of productive uses of electricity (C) |
|  | personal character (P) |
|  | level of entrepreneurial experience (P, C) |
| Enterprise Characteristics | wealth (income, assets, capital) (P, C) |
|  | type/industry (P, C) |
|  | size (P, C) |
|  | registration and formality (P) |
|  | location of enterprise (U, P) |
| Market Access (general) |  |
| Market Access: Demand | population density (U, P, C) |
|  | urban/rural migration (U) |
|  | local wealth and purchasing power (P) |
|  | local population/market size (P) |
|  | number of people who transit (U, P, C) |
|  | level of competition (P) |
| Market Access: Supply/Input factors | access to finance (presence of banks, interest rate, required collateral, required owners’ equity contribution) (U, P, C) |
|  | cost of input factors (other than electricity) (C) |
|  | presence electricity access (U, P) |
|  | electricity access capacity (voltage, AC/DC, …) quality (number and duration of blackouts in given time; presence of risk factors for supply) (U, P, C) |
|  | cost of electrical connection (C) |
|  | cost of electricity consumption (C) |
| Physical Market Access and Transportation | physical access to input goods (e.g. appliances) and services (e.g. maintenance) (P, C) |
|  | level of urbanization (U, P) |
|  | distance to urban centers (U) |
|  | presence of roads and distance to roads (U, P, C) |
|  | quality of roads (P, C) |
|  | presence of regular market/commercial center (U, P) |
|  | access to daily public transportation (U) |
| Governance, institutional context and development | conductive regulatory environment and policies (U, P, C) |
|  | effort to start a business (U, P) |
|  | level of corruption (U, P) |
|  | political relevance and activity of locality (presence of communal administration) (P) |
|  | level of social security (U) |
|  | availability of subsidies to SMEs (U) |
|  | Country (P) |
|  | GDP (U, P) |
|  | gender equality (U) |
|  | presence of violent conflicts (P) |
|  | access to water (P) |
|  | presence of schools (P) |
|  | presence of health facilities (P) |
|  | Investment climate (U) |
|  | access to cell phone communication (P) |
| Other | Season (U, P) |
|  | weather (droughts) (C) |
| Development Program Characteristics | training and information provided and quality (BDS), knowledge sharing (U, P, C) |
|  | monitoring progress and satisfaction (P, C) |

Table 9. All variables found with sources and feasibility of modelling

| **Category** | **Predictor** | **Variables found** | **Source** | **Modelling possible?** | **Level** | **Modelling possible per regression** | | | |
| --- | --- | --- | --- | --- | --- | --- | --- | --- | --- |
|  |  |  |  |  |  | **U** | **Conn** | **S** | **Cons** |
| **Household characteristics** | | | | | | | | | |
|  | Income/level of wealth/assets | share of population by wealth quintile | DHS | yes | state | y | y | y | y |
|  |  | value of dwelling and different assets | WB HH survey | no, no data for SSA | HH | n | n | n | n |
|  |  | wealth index gini coefficient | DHS | yes | state | y | y | y | y |
|  |  | income from different sources | WB HH survey | no, no data for SSA | HH | n | n | n | n |
|  |  | value and possession of different assets | WB HH survey | no, no data for SSA | HH | n | n | n | n |
|  |  | share of population who possess different assets | DHS | no, no sufficient representations | state | n | n | n | n |
|  | number of hh members | number of HH members | WB HH survey | yes | HH | y | y | n | n |
|  |  | share of population per household size | DHS | yes | state | y | y | - | - |
|  | number of rooms | number of rooms | WB HH survey | no, no data for SSA | HH | n | n | n | n |
|  | shock experience | dichotomous variables on the occurrence of different shocks on community and hh level | WB HH survey | yes | HH | y | y | n | n |
|  |  | occurrence of droughts | Beguería and Serrano (2016) | yes | grid-cell | y | y | n | n |
|  |  | occurrence of floods | Beguería and Serrano (2016) | yes | grid-cell | y | y | n | n |
|  | house size | - | - | - | - | - | - | - | - |
|  | agricultural activity  (precipitation) | agricultural activity of any household member (dichotomous) | WB HH survey | yes | HH | y | y | n | n |
|  |  | share of population occupied in agriculture (m/f) | DHS | yes | state | y | y | y | y |
|  | access to information (e.g. radio ownership) | share of HH who own a radio | DHS | yes | state | y | y | y | y |
|  |  | indication if a HH owns a radio | WB HH Survey | yes | HH | y | y | y | y |
| **Entrepreneur characteristics** | | | | | | | | | |
|  | knowledge/skills (general) | - | - | - | - | - | - | - | - |
|  | level of education (years of schooling, literacy) | attendance of any school (dichotomous) | WB HH survey | yes | individual | y | y | n | n |
|  |  | Highest level of education of top manager | WB EP Survey | no, too many missing values | EP | n | n | n | n |
|  |  | ability to read and write in any language | WB HH survey | yes | individual | y | y | n | n |
|  |  | share of population by highest level of schooling (categorical) (m/f) | DHS | yes | state | y | y | y | y |
|  |  | share of literate population (m/f) | DHS | yes | state | y | y | y | y |
|  | digital and mechanical know-how | indication if a HH owns a mobile phone | WB HH Survey | yes | HH | y | y | n | n |
|  |  | share of population who use a mobile phone for financial transactions | DHS | yes | state | y | y | y | y |
|  |  | share of population who owns a mobile phone | DHS | yes | state | y | y | y | y |
|  |  | share of households who own a mobile phone | DHS | yes | state | y | y | y | y |
|  |  | share of households who own a computer | DHS | yes | state | y | y | y | y |
|  |  | internet usage by frequency (m/f) | DHS | yes | state | y | y | y | y |
|  | social networks (religion) | religion | WB HH Survey | yes | individual | y | y | n | n |
|  | marital status | share of men married or living in union | DHS | no, no data for SSA | state | n | n | n | n |
|  |  | share of women married or living in union | DHS | no, no data for SSA | state | n | n | n | n |
|  |  | marital status (categorical) | WB HH survey | no, no data for SSA | individual | n | n | n | n |
|  | age | age of HH head | WB HH Survey | no, no data for SSA | individual | n | n | n | n |
|  |  | age of manager | WB HH Survey | no, no data for SSA | individual | n | n | n | n |
|  |  | share of population per age groups in 10-year steps | DHS | yes | state | y | y | y | y |
|  | sex | any female owners | WB EP Survey | no, too many missing values | EP | n | n | n | n |
|  |  | sex of manger | WB HH Survey | yes | EP | n | y | n | n |
|  |  | sex of HH head | WB HH Survey | yes | HH | y | y | n | n |
|  |  | share of female HH heads | DHS | yes | state | y | y | y | y |
|  | awareness and acceptance of productive uses of electricity | - | - | - | - | - | - | - | - |
|  | level of entrepreneurial experience | years of experience of the top manager | WB EP Survey | no, no data for SSA | EP | n | n | n | n |
| **Enterprise Characteristics** | | | | | | | | | |
|  | wealth (income, assets, capital) | total sales of the last fiscal year of the enterprise (in LCU) | WB EP Surveys | yes | EP | n | n | - | y |
|  |  | total sales in last financial year (Naira) | WB HH Surveys | yes | EP | - | y | n | n |
|  |  | value of different assets | WB EP Surveys | no, no data for SSA | EP | n | n | n | n |
|  | type/industry | 2-digit ISIC 4 | WB HH Survey | yes | EP | y | y | n | n |
|  |  | 4-digit ISIC 3.1 | WB EP Survey | yes | EP | n | n | y | y |
|  | size | number of full-time employees | WB EP Survey | no, no data for SSA | EP | n | n | n | n |
|  | registration and formality | registration status | WB EP Survey | no, no data for SSA | EP | n | n | n | n |
|  |  | registration status | WB HH Survey | no, no data for SSA | EP | n | n | n | n |
|  | location of enterprise | location of business at home of owner | WB EP Survey | no, no data for SSA | EP | n | n | n | n |
| **Market Access (general)** | | | | | | | | | |
| **Market Access: Demand** | | | | | | | | | |
|  | population density | Settlement type | Florczyk et al. (2019) | yes | grid-cell | y | y | n | n |
|  |  | population density | WB HH Survey | yes | grid-cell | y | y | n | n |
|  | urban/rural migration | - | - | - | - | - | - | - | - |
|  | local wealth and purchasing power | share of population by wealth quintile | DHS | yes | state | y | y | y | y |
|  |  | wealth index gini coefficient | DHS | yes | state | y | y | y | y |
|  | local population/market size | size of locality (categorical) | WB EP Surveys | no, too many missing values | regional | n | n | n | n |
|  | number of people who transit | - | - | - | - | - | - | - | - |
|  | level of competition | - | - | - | - | - | - | - | - |
| **Market Access: Supply/Input factors** | | |  |  |  |  |  |  |  |
|  | access to finance (presence of banks, interest rate, required collateral, required owners’ equity contribution) | share of population with bank account (m/f) | DHS | yes | state | y | y | y | y |
|  |  | Firms using banks to finance investment (% of firms) | WB Development Indicators | yes | national | n | n | y | y |
|  |  | Firms using banks to finance working capital (% of firms) | WB Development Indicators | yes | national | n | n | y | y |
|  |  | Lending interest rate (%) | WB Development Indicators | yes | national | n | n | y | y |
|  |  | community access to bank | WB HH Survey | no, no data for SSA | community | n | n | n | n |
|  |  | community access to microfinance institution | WB HH Survey | no, no data for SSA | community | n | n | n | n |
|  |  | experience of HH with borrowing money | WB HH Survey | no, no data for SSA | HH | n | n | n | n |
|  | cost of input factors (other than electricity) | - | - | - | - | - | - | - | - |
|  | presence electricity access | share of HH with electricity | DHS | yes | state | n | n | y | y |
|  |  | share of population with electricity | DHS | yes | state | n | n | n | n |
|  |  | national score of the Doing Business Indicator for getting an electrical connection | WB Doing Business | yes | national | n | n | y | y |
|  |  | access to generator | WB HH Surveys | no, no data for SSA | HH | n | n | n | n |
|  |  | possession of generator in last FY | WB EP Surveys | no, no data for SSA | EP | n | n | n | n |
|  |  | availability of electricity in HH | WB HH Survey | yes | HH | y | - | n | n |
|  |  | nighttime lights | Elvidge et al. (2017) | yes | grid-cell | y | n | n | n |
|  |  | Access to electricity, urban (% of urban population) | WB Development Indicators | yes | national | n | n | y | y |
|  |  | Access to electricity, rural (% of rural population) | WB Development Indicators | yes | national | n | n | y | y |
|  |  | Access to electricity (% of population) | WB Development Indicators | yes | national | n | n | y | y |
|  |  | available sources of electricity and main source | WB HH Surveys | no, no data for SSA | HH | n | n | n | n |
|  | electricity access capacity (voltage, AC/DC, etc.) quality (number and duration of blackouts in given time; presence of risk factors for supply) | Power outages in firms in a typical month (number) | WB Development Indicators | yes | national | n | n | y | y |
|  |  | Value lost due to electrical outages (% of sales for affected firms) | WB Development Indicators | yes | national | n | n | y | y |
|  |  | Firms experiencing electrical outages (% of firms) | WB Development Indicators | yes | national | n | n | y | y |
|  |  | Electric power transmission and distribution losses (% of output) | WB Development Indicators | no, too many missing values | national | n | n | n | n |
|  |  | experience of outages in last FY (dichotomous) | WB EP Surveys | yes | EP | n | n | y | y |
|  |  | number of outages in an average month in last FY | WB EP Surveys | yes | EP | n | n | y | y |
|  |  | duration of average outage FY | WB EP Surveys | no, no data for SSA | EP | n | n | n | n |
|  | cost of electrical connection | getting electricity cost | WB Doing Business | yes | national | n | n | y | y |
|  | cost of electricity consumption | national and state electricity tariffs | different sources (see section) | yes | national/  state (only Nigeria) | n | n | y | y |
| **Physical Market Access and Transportation** | | | | | |  |  |  |  |
|  | physical access to input goods (e.g. appliances) and services (e.g. maintenance) | indication if a HH owns a television | WB HH Survey | yes | HH | y | y | n | n |
|  |  | share of households possessing a refrigerator | DHS | yes | regional | y | y | y | y |
|  |  | share of households possessing a television | DHS | yes | state | y | y | y | y |
|  | level of urbanization | urban cells | Florczyk et al. (2019) | yes | grid-cell | y | y | n | n |
|  |  | area type (categorical) | European Union GHSL Data Package (2019) | yes | grid-cell | y | y | n | n |
|  |  | Sector (rural/urban) | WB HH Survey | yes | grid-cell | y | y | n | n |
|  |  | size of locality (categorical) | WB EP Surveys | no, too many missing values | regional | n | n | n | n |
|  | distance to urban centers | distance to nearest population center (>20.000 inhabitants) (km) | WB HH survey/ WorldCities/ Florczyk et al. (2019) | yes | HH, gird cell | y | y | n | n |
|  |  | travel time to the next city | Weiss et al. (2018) | yes | grid-cell | y | y | n | n |
|  | presence of roads and distance to roads | distance to nearest major road (km) | WB HH Survey/FERMA/ CIESIN et al. (2013) | yes | HH, gird cell | y | y | n | n |
|  |  | road density | CIESIN et al. (2013) | yes | grid-cell | y | y | n | n |
|  | quality of roads | - | - | - | - | - | - | - | - |
|  | presence of regular market/commercial center | distance to nearest key market centers (km) | WB HH survey/USAID FEWSNET/ Florczyk et al. (2019) | yes | HH, gird cell | y | y | n | n |
|  |  | community access to market | WB HH survey | no, no data for SSA | community | - | - | - | - |
|  | access to daily public transportation | community access to bus stops | WB HH Survey | no, no data for SSA | community | - | - | - | - |
| **Governance, institutional context and development** | | | | | | | | | |
|  | conductive regulatory environment and policies | - | - | - | - | - | - | - | - |
|  | effort to start a business | national/state Distance to Frontier score of the Doing Business Indicator for the ease of starting a business | WB Doing Business | yes | national /state (only Nigeria) | y | y | y | y |
|  |  | The rank of ease of starting a business | WB Doing Business | yes | national /state (only Nigeria) | y | y | y | y |
|  |  | The expected number of days it takes to start a business | WB Doing Business | yes | national /state (only Nigeria) | y | y | y | y |
|  |  | The expected number of procedures it takes to start a business | WB Doing Business | yes | national /state (only Nigeria) | y | y | y | y |
|  |  | The expected cost required to start a business (% of income per capita) | WB Doing Business | yes | national /state (only Nigeria) | y | y | y | y |
|  |  | The expected capital required to start a business | WB Doing Business | yes | national /state (onlyy Nigeria) | y | y | y | y |
|  | level of corruption | - | - | - | - | - | - | - | - |
|  | political relevance and activity of locality (presence of communal administration) | - | - | - | - | - | - | - | - |
|  | level of social security | experience of loss of social services in community (dichotomous) | WB HH Survey | no, no data for SSA | community | - | - | - | - |
|  | availability of subsidies to SMEs | - | - | - | - | - | - | - | - |
|  | country | country | WB EP Survey | yes | - | n | n | y | y |
|  | GDP | GDP PPP | WB Development Indicators | yes | national | n | n | y | y |
|  |  | GDP PPP/capita | WB Development Indicators | yes | national | n | n | y | y |
|  |  | GNI PPP | WB Development Indicators | yes | national | n | n | y | y |
|  |  | GNI PPP/capita | WB Development Indicators | yes | national | n | n | y | y |
|  | gender equality | - | - | - | - | - | - | - | - |
|  | presence of violent conflicts | - | - | - | - | - | - | - | - |
|  | access to water | access to water and quality of supply | WB EP survey | no, no data for SSA | EP | n | n | n | n |
|  | presence of schools | presence of schools (nursery, primary, secondary) | WB HH Survey | no, no data for SSA | community | n | n | n | n |
|  | presence of health facilities | presence of health care facility (health center, hospitals, clinics, doctors, midwifes, dentist, pharmacy) | WB HH Survey | no, no data for SSA | community | n | n | n | n |
|  | investment climate | - | - | - | - | - | - | - | - |
|  | access to cell phone communication | - | - | - | - | - | - | - | - |
| **Other** | | | | | | | | | |
|  | season | - | - | - | - | - | - | - | - |
|  | weather (droughts) | precipitation of the wettest month | WB HH Survey, UC Berkeley (2019) | yes | HH, gird cell | y | y | n | n |
|  |  | annual mean temperature | WB HH Survey, UC Berkeley (2019) | yes | HH, gird cell | y | y | n | n |
|  |  | annual precipitation | WB HH Survey, UC Berkeley (2019) | yes | HH, gird cell | y | y | n | n |
|  |  | occurrence of droughts | Beguería and Serrano (2016) | yes | grid-cell | y | y | n | n |
|  |  | occurrence of floods | Beguería and Serrano (2016) | yes | grid-cell | y | y | n | n |
|  |  | mean temperature of the wettest month | WB HH Survey, UC Berkeley (2019) | yes | HH, gird cell | y | y | n | n |
| **Development Program Characteristics** | | | | | | | | | |
|  | training and information provided and quality (BDS), knowledge sharing | - | - | - | - | - | - | - | - |
|  | monitoring progress and satisfaction | - | - | - | - | - | - | - | - |
| **Further potential predictors** | | | | | | | | | |
|  | availability of digital infrastructure and digital know-how | Fixed broadband subscriptions (per 100 people) | WB Development Indicators | yes | national | n | n | y | y |
|  |  | Mobile cellular subscriptions (per 100 people) | WB Development Indicators | yes | national | n | n | y | y |
|  | (alternative) employment opportunities | share of population who worked in the last 12 months | DHS | yes | state | y | y | y | y |
|  |  | Vulnerable employment, total (% of total employment) (modeled ILO estimate) | WB Development Indicators | yes | national | n | n | y | y |
|  | Prevalence of poverty | Poverty headcount ratio at $1.90 a day ((2011) PPP) (% of population) | WB Development Indicators | yes | national | n | n | y | y |
|  |  | Population living in slums (% of urban population) | WB Development Indicators | yes | national | n | n | y | y |
|  |  | Multidimensional poverty headcount ratio (% of total population) | WB Development Indicators | no, too many missing values | national | n | n | n | n |
|  | Economic activity | New businesses registered (number) | WB Development Indicators | no, too many missing values | national | n | n | n | n |
|  |  | New business density (new registrations per 1,000 people ages 15-64) | WB Development Indicators | no, too many missing values | national | n | n | n | n |
|  | Energy intensity of an economy (maturity and efficiency) | GDP per unit of energy use (constant (2017) PPP $ per kg of oil equivalent) | WB Development Indicators | no, too many missing values | national | n | n | n | n |
|  |  | Investment in energy with private participation (current US$) | WB Development Indicators | no, too many missing values | national | n | n | n | n |
|  |  | Electric power consumption (kWh per capita) | WB Development Indicators | no, too many missing values | national | n | n | n | n |
|  | Other development indicators | Human Development Index | Global Data Lab | yes | state | y | y | n | n |
|  |  | Net ODA received (% of GNI) | WB Development Indicators | yes | national | y | y | n | n |

Table 10. Variables in the Uptake Regression.

| **Category** | **Determinant** | **Available Variables** | **Source** | **Inclusion Status** |  |
| --- | --- | --- | --- | --- | --- |
| **Household Characteristics** | | | | |  |
|  | Income/level of wealth/assets | share of population in the lowest WQ | DHS | final model |  |
|  |  | share of population in the second WQ |  | final model |  |
|  |  | share of population in the middle WQ |  | final model |  |
|  |  | share of population in the fourth WQ |  | final model |  |
|  |  | share of population in the highest WQ |  | excluded |  |
|  |  | largest WQ (categorical) |  | excluded |  |
|  |  | average WQ (scale) |  | excluded |  |
|  |  | wealth index gini coefficient | DHS | excluded |  |
|  | number of hh members | number of HH members | WB HH survey | final model |  |
|  |  | avg. Number of HH members | DHS | for projection |  |
|  | shock experience | occurrence of floods in the past 3 years (dummy) | WB HH survey | final model |  |
|  |  | occurrence of droughts in the past 3 years (dummy) |  | final model |  |
|  |  | occurrence of droughts | (Beguería & Vicente Serrano, 2016) | for projection |  |
|  |  | occurrence of floods | Beguería & Vicente Serrano, 2016) | for projection |  |
|  | agricultural activity  (precipitation) | agricultural activity of any household member (dichotomous) | WB HH survey | final model |  |
|  |  | share of women occupied in agriculture (m/f) | DHS | excluded |  |
|  |  | share of men occupied in agriculture (m/f) |  | excluded |  |
|  |  | share of avg. population occupied in agriculture |  | for projection |  |
|  | access to information (e.g. radio ownership) | share of HH who own a radio | DHS | excluded |  |
|  |  | indication if a HH owns a radio | WB HH Survey | excluded |  |
| **HH Head Characteristics** | | | | |  |
|  | level of education (years of schooling, literacy) | attendance of any school (dichotomous) | WB HH survey | final model |  |
|  |  | ability to read and write in any language | WB HH survey | excluded |  |
|  |  | share of women with secondary or higher education | DHS | excluded |  |
|  |  | share of men with secondary or higher education |  | excluded |  |
|  |  | share of avg. population with secondary or higher education |  | excluded |  |
|  |  | share of women with no education | DHS | excluded |  |
|  |  | share of men with no education |  | excluded |  |
|  |  | share of avg. population with no education |  | excluded |  |
|  |  | share women who attended any school | DHS | excluded |  |
|  |  | share of men who attended any school |  | excluded |  |
|  |  | share population who attended any school |  | for projection |  |
|  |  | share of literate women | DHS | excluded |  |
|  |  | share of literate men |  | excluded |  |
|  |  | share of literate avg. population |  | excluded |  |
|  | digital and mechanical know-how | indication if a HH owns a mobile phone | WB HH Survey | final model |  |
|  |  | share of women who use a mobile phone for financial transactions | DHS | excluded |  |
|  |  | share of men who use a mobile phone for financial transactions |  | excluded |  |
|  |  | share of overall population who use a mobile phone for financial transactions |  | excluded |  |
|  |  | share of women who own a mobile phone | DHS | excluded |  |
|  |  | share of men who own a mobile phone |  | excluded |  |
|  |  | share of overall population who owns mobile phone |  | excluded |  |
|  |  | share of households who own a mobile phone | DHS | for projection |  |
|  |  | share of households who own a computer | DHS | excluded |  |
|  |  | women who ever used the internet | DHS | excluded |  |
|  |  | men who ever used the internet |  | excluded |  |
|  |  | avg. population who ever used the internet |  | excluded |  |
|  | age | average age of population | DHS | excluded |  |
|  |  | share of population aged 30-49 |  | excluded |  |
|  |  | sex of HH head | WB HH Survey | final model |  |
|  |  | share of female HH heads | DHS | for projection |  |
|  | social network (religion) | religion | WB HH Survey | excluded |  |
| **Market Access: Demand** | | | | |  |
|  | population density | settlement type dummies | European Union GHSL Data Package (2019) | Final model |  |
|  |  | Population density | WB HH Survey | Excluded |  |
|  |  |  |  |  |  |
| **Market Access: Supply/Input factors** | | | | |  |
|  | access to finance (presence of banks, interest rate, required collateral, required owners’ equity contribution) | share of women with bank account | DHS | excluded |  |
|  |  | share of men with bank account |  | excluded |  |
|  |  | share of overall population with bank account |  | excluded |  |
|  | presence electricity access | share of HH with electricity | DHS | for projection |  |
|  |  | share of population with electricity | DHS | excluded |  |
|  |  | nighttime lights | Elvidge et al. (2017) | final model |  |
|  |  | availability of electricity in HH | WB HH Survey | final model |  |
|  | cost of electricity consumption | national and regional electricity tariffs | different sources (see section) | excluded |  |
| **Physical Market Access and Transportation** | | | | |  |
|  | physical access to input goods (e.g. appliances) and services (e.g. maintenance) | indication if a HH owns a television | WB HH Survey | final model |  |
|  |  | share of households possessing a refrigerator | DHS | excluded |  |
|  |  | share of households possessing a television | DHS | for projection |  |
|  | level of urbanization | urban cells | Florczyk et al. (2019) | final model |  |
|  |  | Sector (rural/urban) | WB HH Survey | final model |  |
|  | distance to urban centers | distance to nearest population center (>20.000 inhabitants) (km) | WB HH survey/WorldCities | final model |  |
|  |  | travel time to the next city | Weiss et al. (2018) | final model |  |
|  | presence of roads and distance to roads | distance to nearest major road (km) | WB HH survey/FERMA | final model |  |
|  |  | road density | CIESIN et al. (2013) | excluded |  |
|  | presence of regular market/commercial center | distance to nearest key market centers (km) | WB HH survey/USAID FEWSNET | final model |  |
| **Governance, institutional context and development** | | | | |  |
|  |  | Human Development Index | Global Data Lab | excluded/highly correlated |  |
|  | effort to start a business | national/regional Distance to Frontier score of the Doing Business Indicator for the ease of starting a business | WB Doing Business | final model |  |
|  |  | The rank of ease of starting a business | WB Doing Business | excluded |  |
|  |  | The expected number of days it takes to start a business | WB Doing Business | excluded |  |
|  |  | The expected number of procedures it takes to start a business | WB Doing Business | excluded |  |
|  |  | The expected cost required to start a business | WB Doing Business | excluded |  |
|  |  | The expected capital required to start a business | WB Doing Business | excluded |  |
|  | weather (droughts) | precipitation of the wettest month | WB HH Survey | excluded |  |
|  |  | annual mean temperature | WB HH Survey | excluded |  |
|  |  | annual precipitation | WB HH Survey | excluded |  |
|  |  | mean temperature of the wettest month | WB HH Survey | excluded |  |
| **Further potential predictors** | | | | |  |
|  | (alternative) employment opportunities | share of women who worked in the last 12 months and are currently | DHS | final model |  |
|  |  | share of men who worked in the last 12 months and are currently |  | final model |  |
|  |  | share of avg. population who worked in the last 12 months and are currently |  | excluded |  |

Table 11. Variables tested for the connection regression.

| **Category** | **Determinant** | **Available Variables** | **Source** | **Inclusion Status** |
| --- | --- | --- | --- | --- |
| **Household Characteristics** | | | | |
|  | Income/level of wealth/assets | share of population in the lowest WQ | DHS | excluded |
|  |  | share of population in the second WQ |  | excluded |
|  |  | share of population in the middle WQ |  | excluded |
|  |  | share of population in the fourth WQ |  | excluded |
|  |  | share of population in the highest WQ |  | final model |
|  |  | largest WQ (categorical) |  | excluded |
|  |  | average WQ (scale) |  | excluded |
|  |  | wealth index gini coefficient | DHS | excluded |
|  | number of hh members | number of HH members | WB HH survey | excluded |
|  |  | avg. Number of HH members | DHS | excluded |
|  | shock experience | occurrence of floods in the past 3 years (dummy) | WB HH survey | excluded |
|  |  | occurrence of droughts in the past 3 years (dummy) |  | final model |
|  |  | occurrence of droughts | yes | for projection |
|  |  | occurrence of floods | yes | excluded |
|  | agricultural activity  (precipitation) | agricultural activity of any household member (dichotomous) | WB HH survey | final model |
|  |  | share of women occupied in agriculture (m/f) | DHS | excluded |
|  |  | share of men occupied in agriculture (m/f) |  | excluded |
|  |  | share of avg. population occupied in agriculture |  | for projection |
| **Entrepreneur Characteristics** | | | | |
|  | level of education (years of schooling, literacy) | attendance of any school (dichotomous) | WB HH survey | excluded |
|  |  | ability to read and write in any language | WB HH survey | final model |
|  |  | share of women with secondary or higher education | DHS | excluded |
|  |  | share of men with secondary or higher education |  | excluded |
|  |  | share of avg. population with secondary or higher education |  | excluded |
|  |  | share of women with no education |  | excluded |
|  |  | share of men with no education |  | excluded |
|  |  | share of avg. population with no education |  | excluded |
|  |  | share women who attended any school | DHS | excluded |
|  |  | share of men who attended any school |  | excluded |
|  |  | share population who attended any school |  | excluded |
|  |  | share of literate women | DHS | excluded |
|  |  | share of literate men |  | excluded |
|  |  | share of literate avg. population |  | excluded |
|  | digital and mechanical know-how | indication if a HH owns a mobile phone | WB HH Survey | final model |
|  |  | share of women who use a mobile phone for financial transactions | DHS | excluded |
|  |  | share of men who use a mobile phone for financial transactions |  | excluded |
|  |  | share of overall population who use a mobile phone for financial transactions |  | excluded |
|  |  | share of women who own a mobile phone | DHS | excluded |
|  |  | share of men who own a mobile phone |  | excluded |
|  |  | share of overall population who owns mobile phone |  | excluded |
|  |  | share of households who own a mobile phone | DHS | for projection |
|  |  | share of households who own a computer | DHS | excluded |
|  | social networks | women who ever used the internet | DHS | excluded |
|  |  | men who ever used the internet |  | excluded |
|  |  | avg. population who ever used the internet |  | excluded |
|  | age | average age of population | DHS | excluded |
|  |  | share of population aged 30-49 |  | excluded |
|  | sex | sex of manager | WB HH Survey | excluded |
|  |  | sex of HH head | WB HH Survey | excluded |
|  |  | share of female HH heads | DHS | excluded |
|  | religion | religion | WB HH Survey | excluded |
| **Enterprise Characteristics** | | | | |
|  | wealth (income, assets, capital) | natural logarithm of total sales of the last fiscal year of the enterprise (USD 2020) | WB HH Surveys | final model |
|  | type/industry | 2-digit ISIC 4 | WB HH Survey | excluded |
| **Market Access: Demand** | | | | |
|  | population density | aera type dummies | European Union GHSL Data Package (2019) | excluded |
|  |  | Population density | WB HH Survey | excluded |
| **Market Access: Supply/Input factors** | | | | |
|  | physical access to input goods (e.g. appliances) and services (e.g. maintenance) | indication if a HH owns a television | WB HH Survey | excluded |
|  |  | share of households possessing a refrigerator | DHS | excluded |
|  |  | share of households possessing a television | DHS | excluded |
|  | access to finance (presence of banks, interest rate, required collateral, required owners’ equity contribution) | share of women with bank account | DHS | excluded |
|  |  | share of men with bank account |  | excluded |
|  |  | share of overall population with bank account |  | excluded |
|  | cost of electricity consumption | national and regional electricity tariffs | different sources (see section) | excluded |
| **Physical Market Access and Transportation** | | | | |
|  | level of urbanization | urban cells | Florczyk et al. (2019) | final model |
|  |  | Sector (rural/urban) | WB HH Survey | excluded |
|  | distance to urban centers | distance to nearest population center (>20.000 inhabitants) (km) | WB HH survey/WorldCities | excluded |
|  |  | travel time to the next city | Weiss et al. (2018) | excluded |
|  | presence of roads and distance to roads | distance to nearest major road (km) | WB HH survey/FERMA | excluded |
|  |  | road density | CIESIN et al. (2013) | excluded |
|  | presence of regular market/commercial center | distance to nearest key market centers (km) | WB HH survey/USAID FEWSNET | excluded |
| **Governance, institutional context and development** | | | | |
|  |  | Human Development Index | Global Data Lab | excluded |
|  | effort to start a business | national/regional Distance to Frontier score of the Doing Business Indicator for the ease of starting a business | WB Doing Business | excluded |
|  |  | The rank of ease of starting a business | WB Doing Business | excluded |
|  |  | The expected number of days it takes to start a business | WB Doing Business | excluded |
|  |  | The expected number of procedures it takes to start a business | WB Doing Business | excluded |
|  |  | The expected cost required to start a business | WB Doing Business | excluded |
|  |  | The expected capital required to start a business | WB Doing Business | excluded |
|  | weather (droughts) | precipitation of the wettest month | WB HH Survey | excluded |
|  |  | annual mean temperature | WB HH Survey | excluded |
|  |  | annual precipitation | WB HH Survey | excluded |
|  |  | mean temperature of the wettest month | WB HH Survey | excluded |
| **Further Potential Predictors** | | | | |
|  | (alternative) employment opportunities | share of women who worked in the last 12 months and are currently | DHS | excluded |
|  |  | share of men who worked in the last 12 months and are currently |  | excluded |
|  |  | share of avg. population who worked in the last 12 months and are currently |  | excluded |

Table 12. Variables testes in the sales model.

| **Category** | **Determinant** | **Variables available** | **Source** | **Inclusion** |
| --- | --- | --- | --- | --- |
| **Household Characteristics** | | | | |
|  | Income/level of wealth/assets | share of population in the lowest WQ | DHS | final model |
|  |  | share of population in the second WQ |  | final model |
|  |  | share of population in the middle WQ |  | final model |
|  |  | share of population in the fourth WQ |  | final model |
|  |  | share of population in the highest WQ |  | excluded |
|  |  | largest WQ (categorical) |  | excluded |
|  |  | average WQ (scale) |  | excluded |
|  |  | wealth index gini coefficient | DHS | excluded |
|  | agricultural activity  (precipitation) | share of women occupied in agriculture (m/f) | DHS | excluded |
|  |  | share of men occupied in agriculture (m/f) |  | excluded |
|  |  | share of avg. population occupied in agriculture |  | excluded |
| **Entrepreneur Characteristics** | | | | |
|  | level of education (years of schooling, literacy) | share of women with secondary or higher education | DHS | excluded |
|  |  | share of men with secondary or higher education |  | excluded |
|  |  | share of avg. population with secondary or higher education |  | excluded |
|  |  | share of women with no education |  | excluded |
|  |  | share of men with no education |  | excluded |
|  |  | share of avg. population with no education |  | final model |
|  |  | share women who attended any school |  | excluded |
|  |  | share of men who attended any school |  | excluded |
|  |  | share population who attended any school |  | excluded |
|  |  | share of literate women |  | excluded |
|  |  | share of literate men |  | excluded |
|  |  | share of literate avg. population |  | excluded |
|  | digital and mechanical know-how | share of women who use a mobile phone for financial transactions | DHS | excluded |
|  |  | share of men who use a mobile phone for financial transactions |  | excluded |
|  |  | share of overall population who use a mobile phone for financial transactions |  | excluded |
|  |  | share of women who own a mobile phone | DHS | excluded |
|  |  | share of men who own a mobile phone |  | excluded |
|  |  | share of overall population who owns mobile phone |  | excluded |
|  |  | share of households who own a mobile phone | DHS | excluded |
|  |  | share of households who own a computer | DHS | excluded |
|  |  | women who ever used the internet | DHS | excluded |
|  |  | men who ever used the internet |  |  |
|  |  | avg. population who ever used the internet |  |  |
|  | age | average age of population | DHS | excluded |
|  |  | share of population aged 30-49 |  | excluded |
|  | sex | share of female HH heads | DHS | final model |
| **Enterprise Characteristics** | | | | |
|  | type/industry | 3-digit ISIC 3.1 | WB EP Survey | excluded |
|  |  | 2-digit ISIC 3.1 |  | excluded |
|  |  | categorical ISIC 3.1 |  | excluded |
| **Market Access (general)** | | | | |
| **Market Access: Demand** | | | | |
| **Market Access: Supply/Input factors** | | | | |
|  | access to finance (presence of banks, interest rate, required collateral, required owners’ equity contribution) | share of women with bank account | DHS | excluded |
|  |  | share of men with bank account |  | excluded |
|  |  | share of overall population with bank account |  | excluded |
|  |  | Firms using banks to finance investment (% of firms) | WB Development Indicators | excluded |
|  |  | Firms using banks to finance working capital (% of firms) | WB Development Indicators | excluded |
|  |  | Lending interest rate (%) | WB Development Indicators | final model |
|  | presence electricity access | share of HH with electricity | DHS | excluded |
|  |  | share of population with electricity | DHS | excluded |
|  |  | Access to electricity, urban (% of urban population) | WB Development Indicators | excluded |
|  |  | Access to electricity, rural (% of rural population) | WB Development Indicators | excluded |
|  |  | Access to electricity (% of population) | WB Development Indicators | excluded |
|  |  | regional score of the Doing Business Indicator for getting an electrical connection | WB Doing Business | final model |
|  | electricity access capacity (voltage, AC/DC, etc.) quality (number and duration of blackouts in given time; presence of risk factors for supply) | Power outages in firms in a typical month (number) | WB PE Survey/ Development Indicators | final model |
|  |  | Value lost due to electrical outages (% of sales for affected firms) | WB Development Indicators | excluded |
|  |  | Firms experiencing electrical outages (% of firms) | WB Development Indicators | excluded |
|  |  | Electric power transmission and distribution losses (% of output) | WB Development Indicators | excluded |
|  |  | experience of outages in last FY (dichotomous) | WB EP Surveys | excluded |
|  | cost of electrical connection | getting electricity cost | WB Doing Business | excluded |
|  | cost of electricity consumption | national and regional electricity tariffs | different sources (see section) | excluded |
| **Physical Market Access and Transportation** | | | | |
|  | physical access to input goods (e.g. appliances) and services (e.g. maintenance) | share of households possessing a refrigerator | DHS | excluded |
|  |  | share of households possessing a television | DHS | excluded |
| **Governance, institutional context and development** | | | | |
|  | effort to start a business | national/regional Distance to Frontier score of the Doing Business Indicator for the ease of starting a business | WB Doing Business | excluded |
|  |  | The rank of ease of starting a business | WB Doing Business | excluded |
|  |  | The expected number of days it takes to start a business | WB Doing Business | excluded |
|  |  | The expected number of procedures it takes to start a business | WB Doing Business | excluded |
|  |  | The expected cost required to start a business | WB Doing Business | excluded |
|  |  | The expected capital required to start a business | WB Doing Business | excluded |
|  | country | SSA region dummy | WB EP Survey | final model |
|  | GDP | GDP PPP | WB Development Indicators | excluded |
|  |  | GDP PPP/capita | WB Development Indicators | excluded |
|  |  | GNI PPP | WB Development Indicators | excluded |
|  |  | GNI PPP/capita | WB Development Indicators | excluded |
| **Further potential predictors** | | | | |
|  | availability of digital infrastructure and digital know-how | Fixed broadband subscriptions (per 100 people) | WB Development Indicators | excluded |
|  |  | Mobile cellular subscriptions (per 100 people) | WB Development Indicators | excluded |
|  | (alternative) employment opportunities | share of population who worked in the last 12 months and are currently | DHS | excluded |
|  |  | Vulnerable employment, total (% of total employment) (modeled ILO estimate) | WB Development Indicators | excluded |
|  | Prevalence of poverty | Poverty headcount ratio at $1.90 a day ((2011) PPP) (% of population) | WB Development Indicators | excluded |
|  |  | Population living in slums (% of urban population) | WB Development Indicators | excluded |
|  | Other development indicators | Human Development Index | Global Data Lab | excluded |
|  |  | Net ODA received (% of GNI) | WB Development Indicators | excluded |

## Appendix 5: Sources of the Variables for the Non-agricultural Enterprise Models

1. World Bank’s Enterprise Surveys: <https://www.enterprisesurveys.org/en/methodology>
2. World Bank Doing Business Indicators:
   - Starting a Business <https://www.doingbusiness.org/en/methodology/starting-a-business>
   - Getting Electricity <https://www.doingbusiness.org/en/methodology/getting-electricity>
3. DHS Program <https://www.dhsprogram.com/What-We-Do/Methodology.cfm>
4. World Development Indicators: <https://datacatalog.worldbank.org/dataset/world-development-indicators>
5. UNDP <http://hdr.undp.org/en/content/human-development-index-hdi>
